# Supplementary figures and images for: Intra-abdominal hypertension and abdominal compartment syndrome in the critically ill liver cirrhotic patient–prevalence and clinical outcomes. A multicentric retrospective cohort study in intensive care
Source: PLoS One. 2021 May 13;16(5):e0251498. doi: 10.1371/journal.pone.0251498 (PMC8118291; doi:10.1371/journal.pone.0251498)

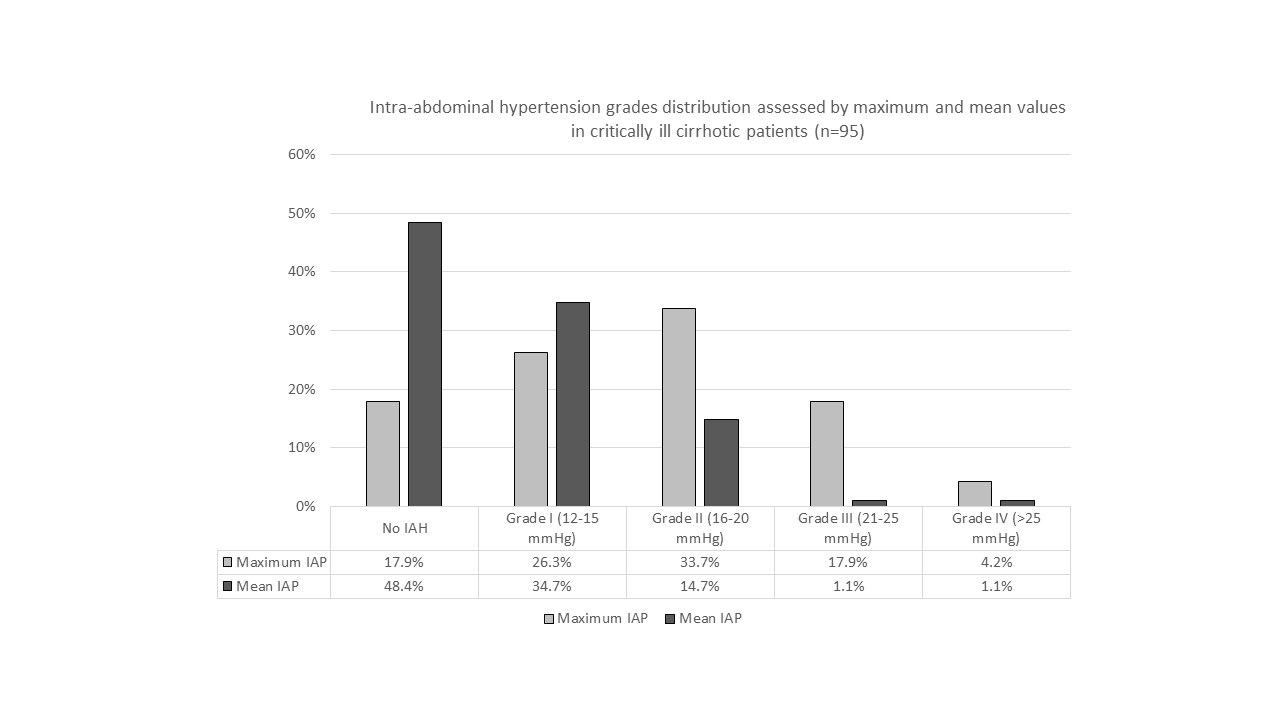

Supplement: S1 Fig — Abbreviations: IAH–Intraabdominal Hypertension; IAP–Intra-abdominal Pressure. (TIF) [file pone.0251498.s001.tif]

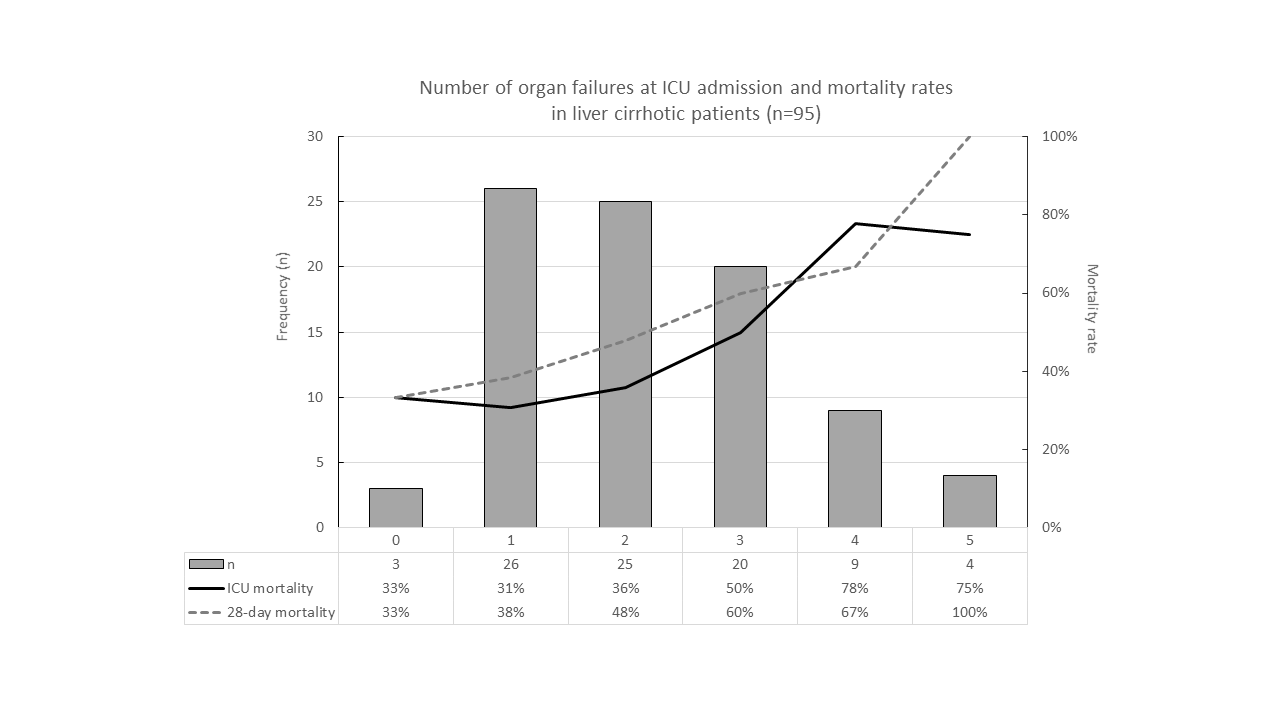

Supplement: S2 Fig — Abbreviations: OF–organ failure; ICU–intensive care unit. (TIF) [file pone.0251498.s002.tif]
