# Supplementary material for: Intra-abdominal hypertension and abdominal compartment syndrome in the critically ill liver cirrhotic patient–prevalence and clinical outcomes. A multicentric retrospective cohort study in intensive care
Source: PLoS One. 2021 May 13;16(5):e0251498. doi: 10.1371/journal.pone.0251498 (PMC8118291; doi:10.1371/journal.pone.0251498)
Supplement: S1 File — List of collaborators from the AbSeS study. (DOCX) [file pone.0251498.s003.docx]

**S1 File. Collaborators AbSeS study**

**National Coordinators:**

**Algeria:** Amin Lamrous (CHU Alger), **Argentina:** Cecilia Pereyra (Hospital Interzonal Agudos Prof Dr Luis Guemes, Buenos Aires), Fernando Lipovestky (Universidad Abierta Interamericana Hospital, Buenos Aires); **Australia**: Despoina Koulenti (UQCCR, Faculty of Medicine, The University of Queensland, Brisbane);**Belgium**: Jan De Waele (Ghent University Hospital, Ghent); **Canada**: Joao Rezende-Neto (St Michael’s Hospital, Toronto); (**Colombia**: Yenny Cardenas (Hospital Universitario Fundación Santa Fe, Bogotá); **Czech Republic**: Tomas Vymazal (Motol University Hospital, Prague); **Denmark**: Hans Fjeldsoee-Nielsen (Fjeldsoee-Nielsen (Nykoebing Falster Hospital, Nykoebing Falster); **France**: Philippe Montravers (CHU Bichat Claude Bernard, Paris); **Germany**: Matthias Kott (Universitätsklinikum, Schleswig-Holstein, Kiel); **Greece**: Arvaniti Kostoula (Papageorgiou General Hospital, Thessaloniki); **India**: Yash Javeri (Nayati Healthcare, Delhi); **Italy**: Massimo Girardis (University Hospital of Modena, Modena); **Israel**: Sharon Einav (Shaare Zedek Medical Centre, Jerusalem); **Netherlands**: Dylan de Lange (University Medical Center, Utrecht); **Peru**: Luis Daniel Umezawa Makikado (Clínica Ricardo Palma, Lima); **Poland**: Adam Mikstacki (Regional Hospital, Poznan); **Portugal**: José-Artur Paiva (Centro Hospitalar Universitário Sao João, Porto); **Romania**: Dana Tomescu (Fundeni Clinical Institute, Bucharest); **Russian Federation**: Alexey Gritsan (Krasnoyarsk State Medical University, Krasnoyarsk Regional Clinical Hospital, Krasnoryarsk); **Serbia**; Bojan Jovanovic (Clinical Center of Serbia, Belgrade); **Singapore**: Kumaresh Venkatesan (Khoo Teck Puat Hospital, Singapore); **Slovenia**: Tomislav Mirkovic (University Medical Centre, Ljubljana); **Spain**: Emilio Maseda (Hospital Universitario La Paz, Madrid); **Turkey**: Yalim Dikmen (Istanbul University-Cerrahpasa, Cerrahpasa Medical School, Istanbul); **United Kingdom**: Benedict Creagh-Brown (Royal Surrey County Hospital NHS Foundation Trust, Guilford);

**AbSeS Investigators:**

**ALGERIA**: CHU (Algiers): Amin Lamrous;

**ARGENTINA:** Sanatorio Güemes (Buenos Aires): Monica Emmerich, Mariana Canale; Sanatorio de la Trinidad Mitre (Buenos Aires): Lorena Silvina Dietz, Santiago Ilutovich; Hospital General de Agudos "Dr. Teodoro Alvarez" (Buenos Aires): John Thomas Sanchez Miñope, Ramona Baldomera Silva; Hospital Militar Central (Buenos Aires): Martin Alexis Montenegro, Patricio Martin; Policlinico Central Union Obrera Metalurgica (Buenos Aires): Pablo Saul, Viviana Chediack; Sanatorio San José (Buenos Aires): Giselle Sutton, Rocio Couce; Hospital General de Agudos "Dr. Ignacio Pirovano" (Buenos Aires): Carina Balasini, Susana Gonzalez; Hospital Britanico (Buenos Aires): Florencia Maria Lascar, Emiliano Jorge Descotte; CMPF Churruca-Visca (Buenos Aires): Natalia Soledad Gumiela, Carina Alejandra Pino; Clinica San Camilo (Buenos Aires): Cristian Cesio, Emanuel Valgolio; Hospital Francisco Javier Muñiz (Buenos Aires): Eleonora Cunto, Cecilia Dominguez; Universidad Abierta Interamericana Hospital (Buenos Aires): Fernando Lipovestky; Hospital Alberto Balestrini (Buenos Aires): Nydia Funes Nelson, Esteban Martin Abegao; Hospital Interzonal Agudos Prof Dr Luis Güemes (Buenos Aires): Cecilia Pereyra, Norberto Christian Pozo; Hospital Español (Buenos Aires): Luciana Bianchi, Enrique Correger; Clinica Zabala (Caba): Maria Laura Pastorino, Erica Aurora Miyazaki; Hospital César Milstein (Caba): Norberto Christian Pozo, Nicolas Grubissich; Hospital Regional Victor Sanguinetti (Comodoro): Mariel Garcia, Natalia Bonetto; Hospital Municipal de Urgencias (Cordoba): Noelia Elizabeth Quevedo, Cristina Delia Gomez; Hospital Manuel B Cabrera (Coronel Pringles): Felipe Queti, Luis Gonzalez Estevarena; Hospital Español de Mendoza (Mendoza,Godoy Cruz: Ruben Fernandez, Ignacio Santolaya; H.I.G.A. Prof. Dr. Luis Güemes (Haedo): Norberto Christian Pozo; Hospital Municipa Doctor Carlos Macias (Mar de Ajo): Sergio Hugo Grangeat, Juan Doglia; Hospital Luis C. Lagomaggiore (Mendoza): Graciela Zakalik, Carlos Pellegrini; Hospital Nacional Profesor Alejandro Posadas (Moron): Maria Monserrat Lloria, Mercedes Esteban Chacon; Hospital Provincial de Neuquen (Neuquen): Mariela Fumale; Clinica Modelo S.A (Paraná): Mariela Leguizamon; Sanatorio de la Ciudad (Puerto Madryn): Irene Beatriz Hidalgo, Roberto Julian Tiranti; Sanatorio Nosti (Rafaela): Paola Capponi, Agustin Tita; Hospital Provincial del Centenario (Rosario): Luis Cardonnet, Lisandro Bettini; Sanatorio Parque (Rosario): Agñel Ramos, Luciano Lovesio; Hospital Papa Francisco (Salta): Edith Miriam Miranda, Angelica Beatriz Farfan; Hospital San Juan Bautista (San Fernando del Valle de Catamarca): Carina Tolosa, Lise Segura; Hospital Central San Isidro Dr Melchor A. Posse (San Isidro-Buenos Aires): Adelina Bellocchio, Brian Alvarez; Hospital Guillermo Rawson (San Juan): Adriana Manzur, Rodolfo Lujan; Establecimiento Asistencial Dr Lucio Molas (Santa Rosa): Natalia Fernandez, Nahuel Scarone; Clínica de Especialidades (Villa María): Alan Zazu, Carina Groh;

**AUSTRALIA:** The Bendigo Hospital (Bendigo): Jason Fletcher, Julie Smith; Coffs Harbour Health Campus (Coffs Harbour): Raman Azad, Nitin Chavan; Concord Hospital (Concord): Helen Wong; Mark Kol; Royal Darwin Hospital (Darwin): Lewis Campbell; Royal Brisbane and Women's Hospital (Herston, Brisbane): Despoina Koulenti, Therese Starr; Sir Charles Gairdner Hospital (Nedlands): Brigit Roberts, Bradley Wibrow; Redcliffe Hospital (Redcliffe): Timothy Warhurst; St Vincent's Hospital (Toowommba): Meher Chinthamuneedi, Bernal Buitrago Ferney;

**BELGIUM:** Cliniques du Sud Luxembourg (CSL)-Hôpital Saint-Joseph (Arlon): Marc Simon; Chirec Hospital (Braine-l’Alleud): Daniel De Backer; Cliniques Universitaires St Luc (Brussels): Xavier Wittebole; Brugmann University Hospital (Brussels): David De Bels, Cliniques de l'Europe - St-Michel (Brussels): Vincent Collin; University Hospital Antwerp (Edegem): Karolien Dams, Philippe Jorens; Ghent University Hospital (Ghent): Jan De Waele; Jessa Ziekenhuis (Hasselt): Jasperina Dubois; University Hospitals Leuven (Leuven): Jan Gunst; CHU Ambroise Paré (Mons): Lionel Haentjens; Clinique Saint-Pierre (Ottignies): Nicolas De Schryver, Thierry Dugernier;

**CANADA**: St. Michael's Hospital (Toronto): Joao Rezende-Neto, Sandro Rizoli

**CHILE**: Hospital Clinico Viña del Mar (Viña del Mar): Paul Santillan

**CHINA**: Jiangsu Province Hospital (Nanjing): Yi Han; Yangpu Hospital of Tongji University (Shanghai): Ewelina Biskup, Changjing Qu; Urumqi General Hospital (Urumqi): Xinyu Li, Wannan Medical College First Affiliated Hospital, Yijishan Hospital (Wuhu): Tao Yu, Lu Weihua;

**COLOMBIA**: Clinica Universitaria Colombia (Bogota): Daniel Molano-Franco, José Rojas, Mederi Hospital (Bogota): Juan Mauricio Pardo Oviedo; Dario Pinilla; Hospital Universitario Fundación Santa Fe (Bogota): Yenny Cardenas, Edgar Celis; Clinica Santa Gracia (Popayan): Mario Arias;

**CROATIA**: Opća bolnica Dubrovnik (Dubrovnik): Anita Vukovic, Maja Vudrag; General Hospital Karlovac (Karlovac): Matija Belavic, Josip Zunic; Clinical Hospital Center Rijeka (Rijeka): Janja Kuharic, Irena Bozanic Kricka; University Hospital Center of Zagreb (Zagreb): Ina Filipovic-Grcic, Boris Tomasevic; University Hospital Center Sestre Milosrdnice (Zagreb): Melanija Obraz, Bruna Bodulica;

**CZECH REPUBLIC**: Nemocnice Břeclav (Břeclav): Martin Dohnal; University Hospital Brno (Brno): Jan Malaska, Milan Kratochvil; Municipal Hospital (Havirov): Igor Satinsky, Peter Schwarz; Hospital Karlovy Vary (Karlovy Vary): Zdenek Kos; University Hospital Olomouc (Olomouc): Ladislav Blahut; University Hospital of Ostrava (Ostrava): Jan Maca; Institute for Clinical and Experimental Medicine (Prague): Marek Protus, Eva Kieslichová;

**DENMARK**: Odense University Hospital (Odense): Louise Gramstrup Nielsen, Birgitte Marianne Krogh

**ECUADOR**: San Vicente de Paúl Hospital (Ibarra): Francisco Rivadeneira; Hospital Oncologico "Dr. Julio Villacreses Colmont" SOLCA (Portoviejo): Freddy Morales, José Mora; Hospital General Puyo (Puyo): Alexandra Saraguro Orozco; Hospital de Especialidades "Eugenio Espejo" (Quito): Diego Rolando MorochoTutillo, Nelson Remache Vargas; Clinica La Merced (Quito): Estuardo Salgado Yepez; Hospital Militar (Quito): Boris Villamagua;

**EGYPT**: Kasr El AINI Hospital, Cairo University (Cairo): Adel Alsisi, Abdelraouf Fahmy;

**FRANCE**: CHU Amiens (Amiens): Hervé Dupont; CHU Angers (Angers): Sigismond Lasocki; Hôpital Beaujon (Clichy): Catherine Paugam-Burtz, Arnaud Foucrier; Centre Hospitalier Compiegne Noyon (Compiègne): Alexandru Nica, Geneviève Barjon; Centre Hospitalier de Lens (Lens): Jihad Mallat; Hôpital Edouard Herriot (Lyon): Guillaume Marcotte; Hôpital Nord (Marseille): Marc Leone, Gary Duclos; Clinique du Millénaire (Montpellier): Philippe Burtin; CHU Bichat Claude Bernard (Paris): Philippe Montravers, Enora Atchade; Groupe Hospitalier Paris Saint-Joseph (Paris): Yazine Mahjoub, Benoît Misset; Hôpital Bichat (Paris) : Jean-François Timsit, Claire Dupuis; CHU de Rouen, Hôpital Charles Nicolle (Rouen): Benoît Veber; Centre Hospitalier Yves le Foll (Saint-Brieuc): Matthieu Debarre; Hôpitaux Universitaires de Strasbourg, NHC -Nouvel Hôpital Civil (Strasbourg): Oliver Collange; Hôpitaux Universitaires de Strasbourg, Hôpital de Hautepierre (Strasbourg): Julien Pottecher, Stephane Hecketsweiler; Hôpital Cochin (Paris): Mélanie Fromentin, Antoine Tesnière;

**GERMANY**: University Hospital Giessen (Giessen): Christian Koch, Michael Sander; Universitätsklinikum Schleswig-Holstein (Kiel): Matthias Kott, Gunnar Elke; University Hospital of Leipzig (Leipzig): Hermann Wrigge, Philipp Simon;

**GREECE**: General Hospital of Agios Nikolaos (Agios Nikolados): Anthoula Chalkiadaki, Charalampos Tzanidakis; Democritus University of Thrace (Alexandroupolis): Ioannis Pneumatikos, Eleni Sertaridou; Evangelismos Hospital (Athens): Zafiria Mastora, Ioannis Pantazopoulos; Hippocrateion General Hospital of Athens (Athens): Metaxia Papanikolaou, Theonymfi Papavasilopoulou; General Hospital Laiko (Athens): John Floros, Virginia Kolonia; University Hospital Attikon (Athens): George Dimopoulos, Chryssa Diakaki; General Hospital Asklepieio Voulas (Athens): Michael Rallis, Alexandra Paridou; General Hospital G. Gennimatas (Athens): Alexandros Kalogeromitros, Vasiliki Romanou; Konstantopouleio Hospital (Athens): Charikleia Nikolaou, Katerina Kounougeri; Agioi Anargiroi General Oncological Hospital of Kifissia (Athens): Evdoxia Tsigou, Vasiliki Psallida; Red Cross Hospital (Athens): Niki Karampela, Konstantinos Mandragos; General Hospital St George (Chania): Eftychia Kontoudaki, Alexandra Pentheroudaki; Thriassio General Hospital of Eleusis (Eleusis): Christos Farazi-Chongouki, Agathi Karakosta; Giannitsa General Hospital (Giannitsa): Isaac Chouris, Vasiliki Radu; University Hospital Heraklion (Heraklion): Polychronis Malliotakis, Sofia Kokkini; Venizelio General Hospital of Heraklion (Heraklion): Eliana Charalambous, Aikaterini Kyritsi; University Hospital of Ioannina (Ioannina): Vasilios Koulouras, Georgios Papathanakos; General Hospital Kavala (Kavala): Eva Nagky, Clairi Lampiri; Lamia General Hospital (Lamia): Fotios Tsimpoukas, Ioannis Sarakatsanos; Agios Andrea's General Hospital of Patras (Patras): Panagiotis Georgakopoulos, Ifigeneia Ravani; Tzaneio General Hospital (Pireaus): Athanasios Prekates, Konstantinos Sakellaridis; General Hospital of Pyrgos (Pyrgos Hleias): Christos Christopoulos, Efstratia Vrettou; General Hospotal of Rethymnon (Rethymnon): Konstantinos Stokkos, Anastasia Pentari; Papageorgiou Hospital (Thessaloniki): Kostoula Arvaniti, Kyriaki Marmanidou; Hippokration Hospial (Thessaloniki): Christina Kydona, Georgios Tsoumaropoulos; G. Papanikolaou General Hospital (Thessaloniki): Militisa Bitzani, Paschalina Kontou; Agios Pavlos Hospital (Thessaloniki): Antonios Voudouris, Elli-Nikki, Flioni; General Hospital of Thessaloniki G.Gennimatas (Thessaloniki): Elli Antypa, Eleftheria Chasou; Theagenio Anticancer Hospital (Thessaloniki): Souzana Anisoglou, Eirini Papageorgiou; General Hospital of Trikala (Trikala): Theoniki Paraforou, Agoritsa Tsioka; Achillopoyleio General Hospital Volos (Volos): Antigoni Karathanou; Xanthi General Hospital (Xanthi): Aristeidis Vakalos;

**INDIA**: CIMS Hospital (Ahmedabad): Bhagyesh Shah, Chirag Thakkar; CHL Hospitals (Indore): Nikhilesh Jain; Sanjay Gandhi Postgraduate Institute of Medical Sciences (SGPGIMS) (Lucknow): Mohan Gurjar, Arvind Baronia; Ruby Hall Clinic (Pune): Prachee Sathe, Shilpa Kulkarni; Jubilee Mission Medical College & Research Institute (Thrissur): Cherish Paul, John Paul;

**IRAN**: Nemazi Hospital (Shiraz): Mansoor Masjedi; Anesthesiology and Critical Care Research Center, Shiraz University of Medical Sciences (Shiraz): Reza Nikandish, Farid Zand; Shiraz Trauma Hospital (Shiraz): Golnar Sabetian; Shohada Hospital (Tabriz): Ata Mahmoodpoor; Masih Daneshvari Hospital (NRITLD (Tehran): Seyed Mohammadreza Hashemian;

**ISRAEL**: Hadassah Hebrew University Medical Center (Jerusalem): Miklosh Bala;

**ITALY**: Cardarelli Ospedale (Campobasso): Romeo Flocco, Sergio Torrente; PinetaGrande Private Hospital (Castel Volturno): Vincenzo Pota; Arcispedale Sant'Anna (Ferrara): Savino Spadaro, Carlo Volta; University Hospital of Modena (Modena): Massimo Girardis, Giulia Serafini; Ospedale S.Antonio (Padova): Sabrina Boraso, Ivo Tiberio; Azienda Ospedaliera Universitaria Policlinico Paolo Giaccone (Palermo): Andrea Cortegiani, Giovanni Misseri;  Azienda Ospedaliero-Universitaria di Parma (Parma): Maria Barbagallo, Davide Nicolotti; Azienda Ospedaliero-Universitaria Pisana (Pisa): Francesco Forfori, Francesco Corradi; Fondazione Policlinico Universitario A.Gemelli IRCCS (Roma): Massimo Antonelli, Gennaro De Pascale; Regina Elena National Cancer Institute of Rome (Roma): Lorella Pelagalli; Azienda Ospedaliero-Universitaria Citta della Salute e della Scienza di Torino, Presidio Ospedaliero Molinette (Torino): Luca Brazzi, Ferdinando Giorgio Vittone; Policlinico Universitario GB Rossi (Verona): Alessandro Russo, Davide Simion; University-Hospital of Foggia (Foggia): Antonella Cotoia, Gilda Cinnella

**JAMAICA**: University Hospital of the West Indies (Kingston): Patrick Toppin, Roxanne Johnson-Jackson;

**JAPAN**: Kameda General Hospital (Kamogawa): Yoshiro Hayashi, Ryohei Yamamoto; Japanese Red Cross Musashino Hospital (Tokyo): Hideto Yasuda, Yuki Kishihara; Okinawa Prectural Chube Hospital (Uruma, Okinawa): Junji Shiotsuka;

**MEXICO**: UMAE Hospital Especialidades Antonio Fraga Mouret-Centro Medico Nacional La Raza IMSS (Mexico City): Luis Alejandro Sanchez-Hurtado, Brigitte Tejeda-Huezo; Hospital Juárez de Mexico (Mexico City): Luis Gorordo; Instituto Nacional de Cancerologia (Mexico City): Silvio A. Ñamendys-Silva, Francisco J. Garcia-Guillen; Hospital general # 5 IMSS (Nogales, Sonora): Manuel Martinez; Hospital Regional de Alta Especialidad de la Península de Yucatán (Merida, Yacatan): Erick Romero-Meja, Ever Colorado-Dominguez;

**NETHERLANDS**: Deventer Hospital (Deventer): Huub van den Oever, Karel Martijn Kalff; Medisch Spectrum Twente (Enschede): Wytze Vermeijden, Alexander Daniel Cornet; Tjongerschans Hospital (Heerenveen): Oliver Beck, Nedim Cimic; Zuyderland Medisch Centrum (Heerlen): Tom Dormans, Laura Bormans; Erasmus MC University Medical Center (Rotterdam): Jan Bakker, Ditty Van Duijn; Elisabeth-TweeSteden Ziekenhuis (Tilburg): Gerrit Bosman, Piet Vos; University Medical Center (Utrecht): Dylan de Lange, Jozef Kesecioglu; Diakonessenhuis (Utrecht): Lenneke Haas;

**OMAN**: Khoula Hospital (Muscat): Akram Henein;

**PARAGUAY**: Hospital Regional de Luque (Luque): Ariel M Miranda;

**PERU**: Clínica Ricardo Palma (Lima): Luis Daniel Umezawa Makikado, Gonzalo Ernesto Gianella Malca; Victor Lazarte Echegaray Hospital (Trujillo): Abel Arroyo-Sanchez;

**POLAND**: Silesian Hospital Cieszyn (Cieszyn): Agnieszka Misiewska-Kaczur; Wojewodzki Szpital Zesoloby w Koninie (Konin): Frisch Akinyi; First Public Teaching Hospital (Lublin): Miroslaw Czuczwar; Szpital Wojewodzki w Opolu SPZOZ (Opole): Karolina Luczak; SPZZOZ w Ostrowi Mazowieckiej (Ostrow Mazowiecka): Wiktor Sulkowski; Poznan University of Medical Sciences, Regional Hospital in Poznan (Poznan): Barbara Tamowicz, Adam Mikstacki; Centrum Medyczne (Poznan): Beata Swit, Bronisław Baranowski; University Hospital (Poznan): Piotr Smuszkiewicz, Iwona Trojanowska; WSM im. J. Strusia (Poznan): Stanislaw Rzymski; Niepubliczny Zakład Opieki Zdrowotnej Szpital w Puszczykowie im. prof. Stefana Tytusa Dąbrowskiego (Puszczykowo): Mariusz Sawinski, Marta Trosiak; Infant Jesus Teaching Hospital of Warsaw Medical University (Warsaw): Malgorzata Mikaszewska-Sokolewicz;

**PORTUGAL**: Hospital de Braga (Braga): Ricardo Alves, Dina Leal; Centro Hospitalar Algarve (Faro): Andriy Krystopchuk, Pedro Muguel Hilario Mendonca; Centro Hospitalar Universit**á**rio Lisboa Central - Hospital Curry Cabral (Lisboa): Rui Antunes Pereira; Centro Hospitalar Universit**á**rio Lisboa Norte - Hospital de Santa Maria (Lisboa): Maria Raquel Lopes Marques de Carvalho, Carlos Candeias; Hospital Pedro Hispano (Matosinhos): Elena Molinos, Amélia Ferreira; Centro Hospitalar Sao Joao - Serviço Medicina Intensiva - UCIPU (Porto): Guiomar Castro, José-Artur Paiva; Centro Hospitalar Sao Joao - Serviço Medicina Intensiva - UCIPG (Porto):José-Manuel Pereira; Centro Hospitalar Sao Joao - Infectious Diseases ICU (Porto): Lurdes Santos, Alcina Ferreira; Hospital do Litoral Alentejano (Santiago do Cacém): Dulce Pascoalinho; São Bernardo - Centro Hospitalar Setubal (Setubal): Rosa Ribeiro, Guilherme Domingos; Hospital Vila Franca de Xira (Vila Franca de Xira): Pedro Gomes, David Nora; Centro Hospitalar de Trás-os-Montes e Alto Douro (Vila Real): Rui Pedro Costa, Anabela Santos;

**QATAR**: Hamad Medical Corporation (Doha): Ahmed Subhy Alsheikhly;

**ROMANIA**: Fundeni Clinical Institute (Bucharest): Dana Tomescu, Mihai Popescu; Regional Institute of Oncology (lasi): Ioana Grigoras, Emilia Patrascanu;

**RUSSIAN FEDERATION**: Krasnodar Regional Hospital #2 (Krasnodar): Igor Zabolotskikh, Tatiana Musaeva; Krasnoyarsk State Medical University, Krasnoyarsk Regional Clinical Hospital (Krasnoyarsk): Alexey Gritsan, Denis Gaigolnik; Vishnevsky Institute of Surgery (Moscow): Vladimir Kulabukhov; Privolzhskiy District Medical Center (Nizhniy Novgorod): Vladislav Belskiy; Clinical Hospital # 4 (Perm): Nadezhda Zubareva, Maxim Tribulev;

**SAUDI ARABIA**: International Medical Center (Jeddah): Ahmed Abdelsalam, Ayman Aldarsani; King Faisal Specialist Hospital & Research Centre (Riyadh): Muhammad Al-Khalid; PSMMC (Riyadh): Ghaleb Almekhlafi, Yasser Mandourah;

**SERBIA**: Clinical Centre of Serbia (Belgrade): Bojan Jovanovic, Krstina Doklestic; Clinic for Digestive Surgery (Belgrade): Jelena Velickovic, Dejan Velickovic; Clinical Center Nis, (Nis): Radmilo Jankovic, Anita Vukovic; Oncology Institute of Vojvodina (Sremska Kamenica): Svetlana Skoric-Jokic, Dragana Radovanovic;

**SOUTH AFRICA**: Charlotte Maxeke Johannesburg Academic Hospital (Johannesburg): Guy Richards, Ahmad Alli;

**SPAIN**: Complejo Hospitalario Universitario de Albacete (Albacete): Maria del Carmen Cordoba Nielfa, Rafael Sánchez Iniesta; Parc de Salut Mar (Barcelona): Adela Benítez-Cano Martínez, Carlos Garcia Bernedo; Hospital Delfos (Barcelona): Santiago Alberto Picos Gil; Vall d'Hebron University Hospital  (Barcelona): Xavier Nuvials, Jordi Rello; Hospital Universitario de Basurto (Bilbao): Joseba Gonzalez Garcia, Jose Manuel Garcia Peña;  Hospital General Universitario Santa Lucia (Cartagena): Roberto Jimenez, Luis Herrera; Hospital General Universitari de Castelló (Castelló): Laura Galarza Barrachina, Ignacio Catalan Monzon; Hospital General Universitario de Ciudad Real (Ciudad Real): Francisco Javier Redondo, Ruben Villazala;  Hospital Costa de la Luz (Huelva): Diego Fernando Matallana Zapata, Isabel Maria Villa Lopez; Hospital Universitari de Bellvitge (L´Hospitalet de Llobregat): Gabriel Moreno-Gonzalez, Juan Carlos Lopez-Delgado; Hospital Universitario de Canarias (La Laguna): Jorge Solera Marin; Hospital Universitario Severo Ochoa (Léganes): Purificacion Sanchez-Zamora; Hospital Universitari Arnau de Vilanova (Lleida): Montserrat Vallverdú Vidal; Hospital Quirón Campo de Gibraltar (Cádiz): Jesús Flores González; Hospital Universitarion del Henares (Madrid): Irene Salinas, Cecilia Hermosa; Hospital Universitario La Paz (Madrid): Emilio Maseda; Hospital Clinico San Carlos (Madrid): Fernando Martinez-Sagasti, Sara Domingo-Marín; Central de la Defensa  Gomez Ulla (Madrid): Johanna Abril Victorino; Hospital 12 de Octubre (Madrid): Raquel Garcia-Alvarez, Pablo López-Arcas Calleja; Hospital Universitario de Malaga (Malaga): Maria-Victoria de la Torre-Prados; CHU Ourense (Ourense): Pablo Vidal-Cortes, Lorena del Río-Carbajo; Complejo Hospitalario de Navarra (Pamplona): Javier Izura, Victoria Minguez; Hospital Universitari Mutua Terrassa (Terrassa): Josep Trenado Alvarez, Anna Parera Prous; Complejo Hospitalario de Toledo  (Toledo): Daniel Paz; Hospital Verge de la Cinta (Tortosa): Ferran Roche-Campo; Hospital Clínico Universitario de Valencia (Valencia): Gerardo Aguilar, Javier Belda; Rio Hortega University Hospital (Valladolid): Jesus Rico-Feijoo, Cesat Aldecoa; Hospital Clinico Universitario Lozano Blesa (Zaragoza): Begoña Zalba-Etayo;

**SWITZERLAND**: Kantonsspital Frauenfeld (Frauenfeld): Martin Lang; Alexander Dullenkopf;

**THAILAND**: Faculty of Medicine Vajira Hospital, Navamindradhiraj University (Bangkok): Konlawij Trongtrakul; Anusang Chtsomkasem;

**TURKEY**: Düzce University Hospital (Duzce): Türkay Akbaş; Ankara University School of Medicine (Ankara): Mustafa Necmettin Unal, Menekse Ozcelik; Akdeniz University Medical School (Antalya): Ayca Gumus, Atilla Ramazanoglu; Trakya University Medical Faculty (Edirne): Dilek Memis, Inal Mehmet; Istanbul University-Cerrahpasa, Cerrahpasa Medical School (Istanbul): Yalim Dikmen, Seval Urkmez; Haydarpaşa Numune Training and Research Hospital (Istanbul): Asu Ozgultekin; Istanbul University Cerrahpasa Medical School Hospital (Istanbul): Oktay Demirkiran; Medipol Mega Hospitals Complex (Istanbul): Nesrin Ahu Aslan, Deniz Kizilaslan; Uludag University, School of Medicine (Nilüfer/Bursa): Ferda Kahveci, Nurdan Ünlü; Elazig Training & Research Hospital (Elazig): Zeynep Ozkan;

**UNITED KINGDOM**: Aberdeen Royal Infirmary (Aberdeen): Callum Kaye, Jan Jansen; Antrim Area Hospital (Antrim): Orla O'Neill, Christopher Nutt; Barnet General Hospital, RFL NHS FT (Barnet): Rajeev Jha, Nicolas Hooker; Basingsoke & North Hampshire Hospital (Basingstoke): Irina Grecu, Christina Petridou; Royal Victoria Hospital (Belfast): Murali Shyamsundar, Lia McNamee; Ulster Hospital (Belfast): John Trinder, Samantha Hagan; Belfast City Hospital (Belfast): Catriona Kelly, Jonathon Silversides; Brighton and Sussex University Hospitals (Brighton): Casiano Barrera Groba, Owen Boyd; West Suffolk Hospital NHS Foundation Trust (Bury St Edmunds): Kaushik Bhowmick, Sally Humphreys; Cambridge University Hospitals NHS Foundation Trust and University of Cambridge (Cambridge): Charlotte Summers, Petra Polgarova; Western Sussex NHS Foundation Trust, St Richard’s Hospital (Chichester, West Sussex): Michael Margarson, Justin Dickens; Colchester General Hospital (Colchester): Suzanne Pearson, Elaine Chinery; Altnagelvin Hospital (Derry): Noel Hemmings, Sinead O'Kane; Ninewells Hospital (Dundee): Pauline Austin, Stephen Cole; Medway NHS Foundation Trust (Gillingham): Catherine Plowright, Roberta Box; Queen Elizabeth University Hospital (Glasgow): Christopher Wright, Lorna Young; Royal Surrey County Hospital (Guildford): Ben Creagh-Brown, Laura Montague; Aintree University Hospital (Liverpool): Robert Parker; Ben Morton; Guy’s and St Thomas Hospitals (London): Marlies Ostermann, Julia Bilinska; University Hospital Lewisham (London): Bernd Oliver Rose, Rosie Reece-Anthony; St Georges University Hospitals NHS Foundation Trust (London): Christine Ryan, Mark Hamilton; King's College Hospital (London): Philip Hopkins, Julia Wendon; Luton and Dunstable Hospital (Luton): Giovanni Brescia, Nazia Ijaz; Maidstone and Tunbridge Wells NHS Trust Hospital (Maidstone): James Wood, Michelle George; Prince Charles Hospital (Merthyr Tydfil): Piroska Toth-Tarsoly; Northumbria Specialist Emergency Care Hospital (Newcastle Upon Tyne): Bryan Yates, Maureen Armstrong; Royal Victoria Infirmary (Newcastle Upon Tyne): Carmen Scott, Christine Boyd; Royal Gwent Hospital (Newport): Tamas Szakmany, David Rees; Kings Mill Hospital (Nottingham): Paul Pulak, Mandy Coggon; Royal Oldham Hospital (Oldham): Bhaskar Saha, Linda Kent; Royal Glamorgan Hospital (Pontyclun): Bethan Gibson; Poole Hospital NHS FT ( Poole): Julie Camsooksai, Henrik Reschreiter; East Surrey Hospital (Redhill): Pat Morgan, Sivatharshini Sangaralingham; Conquest Hospital (St Leonards-on-sea): Alastair Lowe, Petr Vondras; Lister Hospital (Stevenage): Sunil Jamadarkhana, Carina Cruz; University Hospital of North Tees (Stockton-on-Tees): Rakesh Bhandary; Sunderland Royal Hospital (Sunderland): Peter Hersey, Julie Furneval; Musgrove Park Hospital (Taunton): Richard Innes, Patricia Doble; Warwick Hospital (Warwick): Ben Attwood, Penny Parsons; Watford General Hospital (Watford): Valerie Page, Xiaobei Zhao; Royal Hampshire County Hospital (Winchester): Irina Grecu, Julian Dalton;

**UNITED ARAB EMIRATES**: Sheikh Khalifa Medical City (Abu Dhabi): Mohammed Hegazy, Yasser Awad;

**UNITED STATES**: Cleveland Clinic (Cleveland): Douglas Naylor, Amanda Naylor; Detroit Medical Center (Detroit): Sarah Lee; University of South Alabama Medical Center (Mobile, AL): Sidney Brevard, Noelle Davis;
