# Supplementary material for: Intra-abdominal hypertension and abdominal compartment syndrome in the critically ill liver cirrhotic patient–prevalence and clinical outcomes. A multicentric retrospective cohort study in intensive care
Source: PLoS One. 2021 May 13;16(5):e0251498. doi: 10.1371/journal.pone.0251498 (PMC8118291; doi:10.1371/journal.pone.0251498)
Supplement: S1 Table — Multivariable backward stepwise logistic regression including alcoholic cirrhosis, PaO2/FiO2, West-Haven hepatic encephalopathy score and Sodium in the initial step. (DOCX) [file pone.0251498.s004.docx]

S1 Table. Multivariable logistic regression for association with intra-abdominal hypertension.

|  |  |  | 95% Confidence interval | |
| --- | --- | --- | --- | --- |
| (n=86) | *p* value | Odds ratio | Inferior | Superior |
| Alcoholic cirrhosis | 0.01 | 9.9 | 1.7 | 57 |
| West- Haven HE | 0.01 | 2.2 | 1.2 | 3.9 |
| PaO2/FiO2 (mmol) | 0.02 | 1.01 | 1.00 | 1.02 |
| Sodium (mmol/L) | 0.08 | 1.1 | 1.0 | 1.2 |

Multivariable backward stepwise logistic regression including alcoholic cirrhosis, PaO2/FiO2, West-Haven hepatic encephalopathy score and Sodium in the initial step.

Abbreviations: FiO2 - fraction of inspired oxygen; GCS - Glasgow coma score; PaO2 - Partial pressure of arterial oxygen.
