# Supplementary material for: Intra-abdominal hypertension and abdominal compartment syndrome in the critically ill liver cirrhotic patient–prevalence and clinical outcomes. A multicentric retrospective cohort study in intensive care
Source: PLoS One. 2021 May 13;16(5):e0251498. doi: 10.1371/journal.pone.0251498 (PMC8118291; doi:10.1371/journal.pone.0251498)
Supplement: S2 Table — Multivariable backward stepwise logistic regression (n = 83), lactate, WBC, PaO2/FiO2, maximum IAP and MELD score in the initial step (bilirubin and INR are included in MELD score and were therefore excluded from this analysis). Abbreviations: FiO2—fraction of inspired oxygen; IAP- intra-abdominal pressure; INR—international normalization ratio; MELD–Model for End-stage Liver Disease; PaO2—partial arterial oxygen pressure; WBC—white blood cell. (DOCX) [file pone.0251498.s005.docx]

S2 Table. Multivariable logistic regression for 28-day mortality risk factor analysis in critically ill liver cirrhotic patients.

|  |  |  | 95% Confidence interval | |
| --- | --- | --- | --- | --- |
| n=83 | *p* value | Odds ratio | Inferior | Superior |
| Lactate (mmol/L) | 0.04 | 1.15 | 1.01 | 1.31 |
| WBC (10x10^9/mL)^ | 0.03 | 1.09 | 1.01 | 1.17 |
| PaO2/FiO2 | 0.03 | 1.01 | 1.00 | 1.01 |
| MELD | 0.001 | 1.11 | 1.05 | 1.18 |

Multivariable backward stepwise logistic regression (n=83), lactate, WBC, PaO2/FiO2, maximum IAP and MELD score in the initial step (bilirubin and INR are included in MELD score and were therefore excluded from this analysis).

Abbreviations: FiO2 - fraction of inspired oxygen; IAP- intra-abdominal pressure; INR - international normalization ratio; MELD – Model for End-stage Liver Disease; PaO2 - partial arterial oxygen pressure; WBC - white blood cell.
