# Supplementary material for: Intra-abdominal hypertension and abdominal compartment syndrome in the critically ill liver cirrhotic patient–prevalence and clinical outcomes. A multicentric retrospective cohort study in intensive care
Source: PLoS One. 2021 May 13;16(5):e0251498. doi: 10.1371/journal.pone.0251498 (PMC8118291; doi:10.1371/journal.pone.0251498)
Supplement: Attachment — Submitted filename: PONE-D-21-05197_reviewer.pdf [file pone.0251498.s007.pdf]

PLOS ONE

Intra-Abdominal Hypertension and Abdominal Compartment Syndrome in the critically ill liver cirrhotic patient - prevalence and clinical outcomes. A multicentric retrospective cohort study in intensive care.

--Manuscript Draft--

**Manuscript Number:**	PONE-D-21-05197
**Article Type:**	Research Article
**Full Title:**	Intra-Abdominal Hypertension and Abdominal Compartment Syndrome in the critically ill liver cirrhotic patient - prevalence and clinical outcomes. A multicentric retrospective cohort study in intensive care.
**Short Title:**	Intra-Abdominal Hypertension and Abdominal Compartment Syndrome in the critically ill liver cirrhotic patient
**Corresponding Author:**	Rui Pereira<br>Hospital Curry Cabral<br>Lisbon, PORTUGAL
**Keywords:**	Cirrhosis; Acute-on-Chronic Liver Failure; Liver Disease; Ascites
**Abstract:**	Background<br><br>Liver cirrhosis and ascites are risk factors for intra-abdominal hypertension (IAH) and abdominal compartment syndrome (ACS), however, data is scarce.<br><br>We aimed to determine the prevalence of IAH/ACS in a population of critically ill cirrhotic patients with acute medical illness in intensive care and to assess clinical outcomes.<br><br>Methods<br><br>This was a multicentric retrospective cohort study including two general ICUs and pooled data from a multicentric study between January 2010 and October 2019.<br><br>Results<br><br>A total of 9,345 patients were screened, and 95 were included in the analysis.<br><br>Mean age was 56.7±1.3 years, and 79% were male. Liver cirrhosis etiology included alcohol in 45.3% and alcohol plus hepatitis C (HCV) in 9.5%. Precipitating events included infection in 26.2% and bleeding in 21.2% of cases. Mean severity score MELD and SAPS were 26.2±9.9 and 48.5±15.3, respectively, at admission.<br><br>The prevalence of IAH/ACS was respectively 82.1% and 23.2% with a mean value of maximum IAP of 16.0±5.7 mmHg and corresponding grades of IAH: absent 17.9%, I 26.3%, II 33.7%, III 17.9%, and IV 4.2%. Independent risk factors for IAH were alcoholic cirrhosis (p=0.01), West-Haven score (p=0.01), and PaO2/FiO2 ratio (p=0.02); and so was MELD (p=0.03) for ACS.<br><br>Twenty-eight-day mortality was 52.6% and independent risk factors were MELD (p=0.001), maximum IAP (p=0.02), white blood cell (WBC) count (p=0.03), PaO2/FiO2 ratio (p=0.03), and admission lactate (p=0.04).<br><br>Conclusions<br><br>This study demonstrates a very high prevalence of IAH/ACS in the critically ill cirrhotic patient in intensive care. Independent risk factors for IAH/ACS and 28-day mortality included alcoholic cirrhosis, hepatic encephalopathy, PaO2/FiO2 ratio, arterial blood lactate, WBC count, MELD score and maximum IAP. Early diagnosis, prevention, and treatment of IAH/ACS might improve outcome in critically ill cirrhotic patients.
**Order of Authors:**	Rui Pereira

Powered by Editorial Manager® and ProduXion Manager® from Aries Systems Corporation

	Maria Buglevsky
	Rui Perdigoto
	Paulo Marcelino
	Faouzi Saliba
	Stijn Blot
	Joel Starkopf
**Additional Information:**	
**Question**	**Response**
**Financial Disclosure**<br><br>Enter a financial disclosure statement that describes the sources of funding for the work included in this submission. Review the submission guidelines for detailed requirements. View published research articles from *PLOS ONE* for specific examples.<br><br>This statement is required for submission and **will appear in the published article** if the submission is accepted. Please make sure it is accurate.<br><br>**Unfunded studies**<br>Enter: *The author(s) received no specific funding for this work.*<br><br>**Funded studies**<br>Enter a statement with the following details:<br>• Initials of the authors who received each award<br>• Grant numbers awarded to each author<br>• The full name of each funder<br>• URL of each funder website<br>• Did the sponsors or funders play any role in the study design, data collection and analysis, decision to publish, or preparation of the manuscript?<br>• **NO** - Include this sentence at the end of your statement: *The funders had no role in study design, data collection and analysis, decision to publish, or preparation of the manuscript.*<br>• **YES** - Specify the role(s) played.<br><br>* typeset	The authors received no specific funding for this work.
**Competing Interests**<br><br>Use the instructions below to enter a	The authors have declared that no competing interests exist.

competing interest statement for this submission. On behalf of all authors, disclose any competing interests that could be perceived to bias this work—acknowledging all financial support and any other relevant financial or non-financial competing interests.<br><br>This statement is required for submission and **will appear in the published article** if the submission is accepted. Please make sure it is accurate and that any funding sources listed in your Funding Information later in the submission form are also declared in your Financial Disclosure statement.<br><br>View published research articles from *PLOS ONE* for specific examples.<br><br>**NO authors have competing interests**<br><br>Enter: *The authors have declared that no competing interests exist.*<br><br>**Authors with competing interests**<br><br>Enter competing interest details beginning with this statement:<br><br>*I have read the journal's policy and the authors of this manuscript have the following competing interests: [insert competing interests here]*<br><br>* typeset	
**Ethics Statement**<br><br>Enter an ethics statement for this submission. This statement is required if the study involved:<br><br>• Human participants<br>• Human specimens or tissue<br>• Vertebrate animals or cephalopods<br>• Vertebrate embryos or tissues<br>• Field research	The study has been approved by the Ethics Committee of Centro Hospital Universitário Lisboa Central, E.P.E.

Powered by Editorial Manager® and ProduXion Manager® from Aries Systems Corporation

Write "N/A" if the submission does not require an ethics statement.

General guidance is provided below. Consult the submission guidelines for detailed instructions. **Make sure that all information entered here is included in the Methods section of the manuscript.**

Format for specific study types

Human Subject Research (involving human participants and/or tissue)

- Give the name of the institutional review board or ethics committee that approved the study
- Include the approval number and/or a statement indicating approval of this research
- Indicate the form of consent obtained (written/oral) or the reason that consent was not obtained (e.g. the data were analyzed anonymously)

Animal Research (involving vertebrate animals, embryos or tissues)

- Provide the name of the Institutional Animal Care and Use Committee (IACUC) or other relevant ethics board that reviewed the study protocol, and indicate whether they approved this research or granted a formal waiver of ethical approval
- Include an approval number if one was obtained
- If the study involved *non-human primates*, add *additional details* about animal welfare and steps taken to ameliorate suffering
- If anesthesia, euthanasia, or any kind of animal sacrifice is part of the study, include briefly which substances and/or methods were applied

Field Research

Include the following details if this study involves the collection of plant, animal, or other materials from a natural setting:

- Field permit number
- Name of the institution or relevant body that granted permission

**Data Availability**<br><br>Authors are required to make all data underlying the findings described fully available, without restriction, and from the time of publication. PLOS allows rare exceptions to address legal and ethical concerns. See the PLOS Data Policy and FAQ for detailed information.<br><br>A Data Availability Statement describing where the data can be found is required at submission. Your answers to this question constitute the Data Availability Statement and **will be published in the article**, if accepted.<br><br>**Important:** Stating 'data available on request from the author' is not sufficient. If your data are only available upon request, select 'No' for the first question and explain your exceptional situation in the text box.<br><br>Do the authors confirm that all data underlying the findings described in their manuscript are fully available without restriction?	No - some restrictions will apply
**Describe where the data may be found in full sentences. If you are copying our sample text, replace any instances of XXX with the appropriate details.**<br><br>• If the data are **held or will be held in a public repository**, include URLs, accession numbers or DOIs. If this information will only be available after acceptance, indicate this by ticking the box below. For example: *All XXX files are available from the XXX database (accession number(s) XXX, XXX.*).<br>• If the data are all contained **within the manuscript and/or Supporting Information files**, enter the following: *All relevant data are within the manuscript and its Supporting Information files.*<br>• If neither of these applies but you are able to provide **details of access elsewhere**, with or without limitations, please do so. For example:	All relevant data are within the manuscript and its Supporting Information files. Additional data can be requested to the corresponding author.

Data cannot be shared publicly because of [XXX]. Data are available from the XXX Institutional Data Access / Ethics Committee (contact via XXX) for researchers who meet the criteria for access to confidential data.<br><br>*The data underlying the results presented in the study are available from (include the name of the third party and contact information or URL).*<br>• This text is appropriate if the data are owned by a third party and authors do not have permission to share the data.<br><br>* typeset	
Additional data availability information:	Tick here if your circumstances are not covered by the questions above and you need the journal's help to make your data available.

Powered by Editorial Manager® and ProduXion Manager® from Aries Systems Corporation

Manuscript
Click here to access/download;Manuscript;Manuscript PLOS One 1.0.docx

Research article

Intra-Abdominal Hypertension and Abdominal Compartment Syndrome in the critically ill liver cirrhotic patient - prevalence and clinical outcomes. A multicentric retrospective cohort study in intensive care.

Intra-Abdominal Hypertension and Abdominal Compartment Syndrome in the critically ill liver cirrhotic patient

List of authors and filiations:

Rui Pereira, MD[1]; Maria Buglevski, MD [2]; Rui Perdigoto, PhD [1]; Paulo Marcelino, PhD [3]; Faouzi Saliba, PhD [4]; Stijn Blot, PhD [5]; Joel Starkopf, PhD [2].

[1] Hospital de Curry Cabral, Centro Hospitalar Universitário Lisboa Central, Portugal.

[2] University of Tartu, Tartu University Hospital, Estonia.

[3] Hospital de Santa Marta, Centro Hospitalar Universitário Lisboa Central, Portugal.

[4] AP-HP Hôpital Paul Brousse, Université Paris Saclay, INSERM unit 1193, Villejuif, France.

[5] Ghent University, Belgium.

Corresponding author: Rui Pereira <rui.pereira@mail.com>

Address for correspondence and reprints: UCIP-7, Hospital de Curry Cabral. Rua da Beneficência, nº 8, 1069-166 Lisboa, Portugal.

Funding: no funding was used for this original research article.

Author's disclosures: all the authors declare to have no financial or ethical conflicts of interest regarding the contents of this manuscript submission.

Abstract

Background: Liver cirrhosis and ascites are risk factors for intra-abdominal hypertension (IAH) and abdominal compartment syndrome (ACS), however, data is scarce.

We aimed to determine the prevalence of IAH/ACS in a population of critically ill cirrhotic patients with acute medical illness in intensive care and to assess clinical outcomes.

Methods: This was a multicentric retrospective cohort study including two general ICUs and pooled data from a multicentric study between January 2010 and October 2019.

Results: A total of 9,345 patients were screened, and 95 were included in the analysis.

Mean age was 56.7±1.3 years, and 79% were male. Liver cirrhosis etiology included alcohol in 45.3% and alcohol plus hepatitis C (HCV) in 9.5%. Precipitating events included infection in 26.2% and bleeding in 21.2% of cases. Mean severity score MELD and SAPS were 26.2±9.9 and 48.5±15.3, respectively, at admission.

The prevalence of IAH/ACS was respectively 82.1% and 23.2% with a mean value of maximum IAP of 16.0±5.7 mmHg and corresponding grades of IAH: absent 17.9%, I 26.3%, II 33.7%, III 17.9%, and IV 4.2%. Independent risk factors for IAH were alcoholic cirrhosis (p=0.01), West-Haven score (p=0.01), and PaO2/FiO2 ratio (p=0.02); and so was MELD (p=0.03) for ACS.

Twenty-eight-day mortality was 52.6% and independent risk factors were MELD (p=0.001), maximum IAP (p=0.02), white blood cell (WBC) count (p=0.03), PaO2/FiO2 ratio (p=0.03), and admission lactate (p=0.04).

Conclusions: This study demonstrates a very high prevalence of IAH/ACS in the critically ill cirrhotic patient in intensive care. Independent risk factors for IAH/ACS and 28-day mortality included alcoholic cirrhosis, hepatic encephalopathy, PaO2/FiO2 ratio, arterial

blood lactate, WBC count, MELD score and maximum IAP. Early diagnosis, prevention, and treatment of IAH/ACS might improve outcome in critically ill cirrhotic patients.

Keywords: Cirrhosis, Acute-on-Chronic Liver Failure, Liver Disease, Ascites.

Introduction

Pathologic increases of intra-abdominal pressure (IAP) are associated with increased morbidity and mortality in the critically ill patient.(1,2)

Intra-abdominal hypertension (IAH) and abdominal compartment syndrome (ACS) are recognized as independent mortality risk factors in several clinical settings, including major abdominal surgery, ruptured abdominal aortic aneurism, major burns, major trauma, acute pancreatitis, and mechanical ventilation.(1,2) The reported prevalence of IAH/ACS in mixed medical and surgical ICU patients reach 59% and 8%, respectively.(3–5)

Patients with advanced liver cirrhosis are at high risk of developing IAH/ACS.(1) Increased portal vein pressure and subsequent intra-abdominal ascites stipulate the need for symptomatic treatment.(6) Ultimately, refractory ascites leads to chronically increased IAP and the need for repeated percutaneous drainage through paracentesis.(7) There is, however, paucity of data regarding the incidence of IAH and ACS in cirrhotic patients with acute critical illness.

The aim of this study was to assess the prevalence of IAH/ACS and its impact on clinical outcomes in the liver cirrhosis patient admitted to intensive care units (ICU) with acute medical illness.

Methods and Materials

This was a multicentric retrospective cohort study of patients admitted to the ICU with advanced chronic liver disease.

Participating centers included two ICUs and pooled data from a multicentric study on intra-abdominal infection/sepsis.(8)

Center one was Hospital de Curry Cabral, Centro Hospitalar Universitário Lisboa Central, in Portugal, with a general ICU, with 21 beds, specializing in liver disease. Standard of care included IAP measurement. Patients were retrospectively screened for eligibility between October 2016 and October 2019.

Center two was Tartu University Hospital at Estonia, with a medical/surgical ICU, with 10 beds, mostly treating emergency patients. Standard of care included IAP measurement. Patients were retrospectively screened for eligibility between 2010 and 2018.

Supplemental patient enrollment was pooled from the “Abdominal Sepsis Study: Epidemiology of Etiology and Outcome” (AbSeS) multicenter, prospective, observational, epidemiological study of adult ICU patients from the ESICM Trials Group Project diagnosed with intra-abdominal infection.(8)

The reporting of this study followed Strengthening the Reporting of Observational Studies in Epidemiology (STROBE) guidelines.

The Institutional Ethics Committees waived the need for individual informed consent for this observational study. All study procedures followed the principles of the Declaration of Helsinki.

Patients with liver cirrhosis were screened for eligibility, and selection was performed using the following inclusion criteria: first ICU admission during the index hospital stay, age ≥18 years, and known diagnosis of liver cirrhosis. The exclusion criteria were ICU admission for surgical reasons and absence of IAP recordings.

Cirrhosis was defined as bridging fibrosis on previous liver biopsy or a composite of clinical signs and laboratory tests, endoscopy, and radiologic imaging.(9)

The severity scores APACHE II and SAPS II as well as specific scores of liver cirrhosis MELD and MELD-Na were calculated at ICU admission, according to the original formulas.(10–15) Acute-on-chronic liver failure (ACLF) syndrome and organ failure (OF) were defined per the European Foundation for the Study of Chronic Liver Failure Consortium (CLIF-C).(16)

IAP measurement methodology, definitions of IAH and ACS, and clinical management of these patients followed updated guidelines established by the World Society of Abdominal Compartment Syndrome (WSACS).(1,17,18) Accordingly, IAH was classified into Grade I, IAP 12–15 mmHg; Grade II, IAP 16–20 mmHg; Grade III, IAP 21–25 mmHg; and Grade IV, IAP >25 mmHg. Abdominal compartment syndrome (ACS) was defined as a sustained IAP >20 mmHg associated with new organ dysfunction/failure. IAP monitoring was performed via trans-bladder measurement technique with zero-pressure reference point set at the phlebostatic axis in the midaxillary line. Clinical management of IAH/ACS was based on these guidelines and included evacuating intraluminal contents and intra-abdominal space-occupying lesions, improving abdominal wall compliance, optimizing fluid administration, and optimizing systemic and regional perfusion. The patient's maximum IAP was determined using the single highest IAP value of all known measurements, and mean IAP calculation used all recorded measurements during the entire ICU stay.

The clinical management of the critically ill liver cirrhosis patients included in this study was performed by intensivists and complied with the evolving guidelines for the treatment of the precipitating event of the clinical decompensation and for the multiorgan system failure support.(7,19,20)

Variables recorded on admission day included demographics, liver cirrhosis etiology, precipitating event of acute illness, arterial blood lactate concentration, vital organ support with vasopressors and mechanical ventilation, and the following clinical severity scores: Acute

Physiology and Chronic Health Evaluation II (APACHE II), Simplified Acute Physiology Score II (SAPS II), Model for End Stage Liver Disease (MELD), Model for End Stage Liver Disease Sodium (MELD-Na), Sequential Organ Failure Assessment (SOFA), and Chronic Liver Failure SOFA (CLIF-SOFA). Renal replacement therapy (RRT) during the ICU stay was also recorded.

All data on patient characteristics were retrieved on site from medical records and collected in an anonymous and protected database.

Statistical analysis described discrete variables through count and percentage and continuous variables using mean (SD) or median and interquartile range (IQR), where appropriate. The number (n) of patients or measurements is indicated when it differed from the universal number.

Etiology of liver cirrhosis was dichotomized to compare between "alcohol alone or combined" versus "all other causes." Similarly, precipitating events of clinical decompensation were computed into "bleeding," "infection," and "other" for mortality risk analysis.

Univariate analysis of continuous variables was performed using the t-test or the Mann-Whitney U test as a nonparametric alternative where appropriate. Chi-square and Fisher's exact tests were used for categorical variables.

Multivariable analysis was performed using variables with p value ≤0.10 in univariable analysis and backward stepwise logistic regression. Variables were excluded from the final models if p value was > 0.10.

Statistical significance was defined as bilateral p value ≤0.05. Statistical analysis was performed using IBM Statistical Package for Social Science (SPSS), version 23 (IBM Corp, North Castle, NY, US).

Results

A total of 9,345 patients were screened for eligibility, and 95 were included in the analysis. A patient flowchart is described in Figure 1.

Baseline characteristics of patients at ICU admission and comparison between survivors and non-survivors at 28 days are shown in Table 1.

Liver cirrhosis etiology included alcohol in 43 (45.3%) cases, alcohol plus Hepatitis C (HCV) in 6 (9.5%), HCV alone in 6 (6.3%), and other causes such as NASH, alfa-1-antitripsin deficiency, and haemochromatosis in 20 (21.0%) cases. In 17 patients (17.9%), the cause remained unspecified.

Comorbidities were reported in 66 patients with a mean Charlson Comorbidity Index (CCI) of 5.4±2.1. The comorbidities included cirrhosis with clinical signs of portal hypertension (86.4%), diabetes mellitus with and without end-organ damage (9.1% and 22.7%, respectively), solid cancer with metastasis (4.5%) and without metastasis (13.6%), hematologic cancer (1.5%) and chronic kidney disease (15.2%).

Precipitating events of decompensated cirrhosis leading to ICU admission were infection (26.2%), bleeding (21.2%), acute kidney injury (3.2%), and hepatic encephalopathy (3.2%).

In 56 patients, the IAPs were repeatedly measured and recorded, with a median 18 (IQR 8–30) measurements per patient and a total of 1,189 measurements. For the remaining 39 patients, single mean and maximum values were reported without reference to the number of measurements performed.

The prevalence of IAH and ACS was, respectively, 82.1% and 23.2%. IAH grade distribution for maximum and mean values is shown in Supplemental Figure 1. Mean value of maximum IAP was 16.0±5.7 mmHg. The IAH severity, according to maximum IAP values, was the following: no IAH, 17.9%; Grade I, 26.3%; Grade II, 33.7%; Grade III, 17.9%; and Grade IV,

4.2%. The mean value of mean IAPs was 12.1±4.1 mmHg, and the severity of IAH was distributed as follows: no IAH, 48.4%; Grade I, 34.7%; Grade II, 14.7%; Grade III, 1.1%; and Grade IV, 1.1%.

Risk factors associated with IAH in the univariable analysis were higher serum sodium levels (p=0.02), higher Glasgow Coma Scale (GCS) (p=0.03), and higher West-Haven (WH) hepatic encephalopathy score (p=0.04), while alcoholic etiology of cirrhosis (p=0.06) and PaO2/FiO2 (p=0.1) nearly reached significant association (Table 2). Multivariable analysis (excluding GCS due to preference for WH encephalopathy score in the cirrhotic patient) revealed that alcoholic cirrhosis (p=0.01), higher WH scores (p=0.01), and higher PaO2/FiO2 ratio (p=0.02) were independently associated with the presence of IAH (Supplemental Table 1).

Abdominal compartment syndrome was significantly associated with MELD and MELD-Na scores (respectively, p=0.03 and p=0.04) compared to other patients with simple IAH. White blood cell count (p=0.08) and infection as precipitant event (p=0.10) nearly reached significant association with ACS (Table 2). Multivariate analysis (n=71), including the aforementioned variables, confirmed MELD score (OR=1.06; 95% CI: 1.00–1.12; p=0.04) as the only independent predictor of ACS.

In-ICU mortality rate was 46.3% (n=44), and overall, 28-day mortality was 52.6% (n=50). Maximum and mean IAPs were significantly higher in non-survivors (p=0.02 and p=0,04, respectively) (Table 1). Patients with ACS had a significant increase in 28-day mortality when compared to those with IAH Grades I–II (77% versus 46%, respectively, p=0.02). (Table 2)

Univariate analysis of 28-day mortality revealed that non-survivors had significantly worse clinical severity scores, except for the SOFA and Charlson Comorbidity Index, and

significantly elevated values for laboratory variables bilirubin, INR, lactate, and WBC at admission, with PaO2/FiO2 nearly reaching significant association.

Maximum IAP was associated with 28-day mortality when adjusted for SAPS II score (OR=1.11; 95% CI: 1.01–1.22; p=0.02 for n=89) but not for MELD nor MELD-Na (respectively, OR=1.06; 95% CI: 0.97–1.15; p=0.2; and OR=1.07; 95% CI: 0.98-1–16; p=0.1 for n=87).

Multivariable analysis including lactate, WBC, PaO2/FiO2, maximum IAP, and MELD score (excluding bilirubin and INR as these variables are included in MELD score) identified MELD (p=0.001), WBC (p=0.03), PaO2/FiO2 (p=0.03), and lactate (p=0.04) as independent predictors of 28-day mortality. Noticeably, maximum IAP did not reach statistical significance in the multivariable model (Supplemental Table 2).

Classifications of IAP grades and the incidence of OF at ICU admission, with matching mortality rates, are depicted in Figures 2, 3, and Supplemental Figure 2, respectively.

Liver transplantation, although not a reason for ICU admission, was performed in 8 (13.6%) of 59 patients during a 28-day follow-up.

Discussion

This is the first multicentric study to analyze the prevalence and outcomes of IAH and ACS in cirrhotic patients admitted in the ICU with acute medical illness.

The prevalence of IAH and ACS were very high, affecting more than three quarters and nearly one quarter, respectively, of this critically ill population with decompensated liver disease, admitted mainly due to sepsis or upper gastrointestinal bleeding.

Different studies have reported the prevalence of IAH in critical care patients from 32.1% to 58.8% and of ACS from 4.2% to 8.2%.(3–5) In a multicentric IAP study of a mixed population of 265 patients, IAH was present in 32.1% and ACS in 4.2% of patients on ICU admission day.(4) The IROI Study, a multicentric prospective study of 491 mixed ICU patients, demonstrated IAH prevalence in 48.9% and ACS in 6.3% of patients during the first 2 weeks of the ICU stay.(5)

The prevalence of IAP in the critically ill cirrhotic patient has been earlier addressed in a post hoc analysis from a single center randomized controlled trial for the use of low-dose hydrocortisone in septic shock.(21,22) In their study, Al-Dorzi et al. observed 61 cirrhotic patients over the first week in the ICU and described IAH in 97% and ACS in 39% of them. Ascites was present in nearly all patients (95.1%). However, as also recognized by the authors, they very likely overestimated the IAH/ACS prevalence since intra-vesical pressure measurements were performed with vesical instillation of a large volume (50–100 mL), and the zero-pressure reference point was set at symphysis pubis. Current guidelines recommend an injection of 25 mL of sterile saline to prevent bladder overdistension and zero-pressure reference set at the phlebostatic axis in the mid-axillary line.(1) Even so, and considering a possible patient selection bias, the prevalence of IAH was remarkably high.

The present study suggests interrelations between severity of hepatic disease, degree of organ failures, and IAH as we observed IAH being independently associated with alcoholic cirrhosis, hepatic encephalopathy WH score, and respiratory dysfunction (PaO2/FiO2 ratio). This is in accordance with previous studies where liver dysfunction (likely due to the formation of ascites), severe acute illness (APACHE II ≥18 points), respiratory dysfunction (mechanical ventilation with positive end-expiratory pressure ≥7 cm), abdominal distension, ileus, positive fluid balance, obesity (body mass index >27 kg/m^2), and abdominal surgery increase the likelihood of IAH development.(4,5)

Finding that IAH is associated with higher hepatic encephalopathy scores deserves attention. Few studies have reported a correlation between IAH and increased intracranial pressure (ICP), even at low levels of IAP.[21] Furthermore, reversible disruption of the blood-brain barrier and increased ischemic mediators, indicating cerebral ischemia due to IAH, have been demonstrated.(23–28) Two distinct pathways by which IAP could be transmitted to the central nervous system have been proposed, according to the Monro-Kellie hypothesis: first, through the venous plexus of the spinal canal and the intracranial veins backflow, and second, due to cranial excursion of the diaphragm causing elevated intrathoracic pressure as well as augmented central venous pressure decreasing venous drainage from the central nervous system via the jugular system.(23,29,30) All these mechanisms could represent an additional pathophysiologic rationale for hepatic encephalopathy in liver disease patients with increased IAP.(23–30)

A higher PaO2/FiO2 was, strikingly, associated with IAH. The reason for this observation was not obvious to us and it could be speculated that higher levels of PEEP may have been used in these mechanically ventilated patients with IAH, thus providing an increase in PaO2, although, available data was could not confirm this.

A high mortality rate was observed and comparable with other studies in critically ill liver cirrhosis patients showing short-term mortality rates ranging from 34.9% to 71.9%.(31–33)

Increased IAP was associated with higher 28-day mortality, particularly with ACS. The severity of IAH was progressively associated with mortality, which was similarly high at IAH Grades III and IV. This observation strengthens the argument for merging these grades together, as suggested by Blaser et al.(5)

Mean levels of IAP presented higher mortality rates than matching levels of maximum IAP. It could be speculated that the deleterious clinical impact of sustained (mean) IAP, more so than episodical (maximum) IAP, plays a predominant role in the ultimate effect and highlights the importance of the duration of IAH and time-dependence of its pathological effects on organ dysfunction.(2)

Multivariable analysis of 28-day mortality identified MELD score, WBC count, Pa/FiO2 ratio, and lactate concentration as independent risk factors, confirming other studies.(16,32,34,35)

Although the IROI study reported the presence and severity of IAH as an independent risk factor for increased mortality in a mixed population of critical patients, our study did not observe this.(5) Similarly, IAH was reported to be associated with renal failure and RRT in septic shock liver disease patients and in those requiring longer ventilatory and vasopressor support.[17] However, we did not observe such associations. A likely explanation is the selection bias since the prevalence of IAH in our study patients was notably high. Further research is needed to confirm these findings.

Clinical practice guidelines for the critically ill patient with IAH/ACS recommend a stepwise medical management algorithm to reduce and maintain IAP ≤15 mmHg. Importantly, ascites stands out as a fundamental common feature in critical liver disease patients with IAH. Studies in intensive care have reported the safety of large volume paracentesis, thus potentially lowering IAP, with beneficial effects on hepatosplenic blood flow as well as respiratory and renal functions.(36–40) Particular relevance should be assigned to the clinical management of cirrhotic patient with ascites, especially given the therapeutic option for paracentesis to treat and potentially prevent the recurrence of IAH and ACS during an ICU stay. A randomized controlled trial on this subject (COPPTRIAHL, NCT04322201) is underway

with the intent to control IAH and improve clinical outcomes and may provide future insights.(41)

External validation of our results is required, although we believe them to be generalizable given the multicentric design of the study and the reasonable number of patients.

Some noteworthy limitations in this study precluded more accurate reporting and interpretation of results. These include valuable variables that were not assessed, such as abdominal perfusion pressure, fluid balance, and in particular therapeutic interventions aimed at lowering IAP, such as abdominal paracentesis. Possible selection and sampling biases were minimized due to the multicentric collection of data at each study site and the reasonably sized patient cohort.

Conclusions

Our study demonstrates a very high incidence of IAH and ACS in the critically ill liver cirrhotic patient with acute medical illness when compared to other populations of mixed intensive care patients. Increased IAP is associated with severity of disease and adverse outcomes. Independent risk factors for IAH/ACS and 28-day mortality include alcoholic cirrhosis, hepatic encephalopathy, PaO2/FiO2 ratio, arterial blood lactate, WBC count, and MELD score.

Particular relevance should be given to early diagnosis, treatment, and prevention of IAH and ACS as it might improve outcome in the liver cirrhosis patient in the ICU.

References

1. Kirkpatrick AW, Roberts DJ, De Waele J, Jaeschke R, Malbrain MLNG, De Keulenaer B, et al. Intra-abdominal hypertension and the abdominal compartment syndrome: Updated consensus definitions and clinical practice guidelines from the World Society of the Abdominal Compartment Syndrome. Intensive Care Med [Internet]. 2013 Jul 15;39(7):1190–206. Available from: http://link.springer.com/10.1007/s00134-013-2906-z
2. Kyoung K-H, Hong S-K. The duration of intra-abdominal hypertension strongly predicts outcomes for the critically ill surgical patients: a prospective observational study. World J Emerg Surg. 2015 Dec;10(1):22.
3. Malbrain MLNG, Chiumello D, Pelosi P, Wilmer A, Brienza N, Malcangi V, et al. Prevalence of intra-abdominal hypertension in critically ill patients: a multicentre epidemiological study. Intensive Care Med [Internet]. 2004 May 1 [cited 2018 Nov 13];30(5):822–9. Available from: http://www.ncbi.nlm.nih.gov/pubmed/14758472
4. Malbrain MLNG, Chiumello D, Pelosi P, Bihari D, Innes R, Ranieri VM, et al. Incidence and prognosis of intraabdominal hypertension in a mixed population of critically ill patients: A multiple-center epidemiological study. Crit Care Med. 2005 Feb;33(2):315–22.
5. Reintam Blaser A, Regli A, De Keulenaer B, Kimball EJ, Starkopf L, Davis WA, et al. Incidence, Risk Factors, and Outcomes of Intra-Abdominal Hypertension in Critically Ill Patients - A Prospective Multicenter Study (IROI Study). Crit Care Med. 2019;47(4):535–42.

6. Moore KP, Wong F, Gines P, Bernardi M, Ochs A, Salerno F, et al. The management of ascites in cirrhosis: Report on the consensus conference of The International Ascites Club. Hepatology. 2003;38(1):258–66.
7. European Association for the Study of the Liver, Ginés P, Angeli P, Lenz K, M??ller S, Moore K, et al. EASL clinical practice guidelines on the management of ascites, spontaneous bacterial peritonitis, and hepatorenal syndrome in cirrhosis. Journal of Hepatology Sep, 2010 p. 397–417.
8. Blot S, Antonelli M, Arvaniti K, Blot K, Creagh-Brown B, de Lange D, et al. Epidemiology of intra-abdominal infection and sepsis in critically ill patients: "AbSeS", a multinational observational cohort study and ESICM Trials Group Project. Intensive Care Med. 2019;
9. Olson JC, Kamath PS. Acute-on-chronic liver failure: concept, natural history, and prognosis. Curr Opin Crit Care [Internet]. 2011 Apr [cited 2017 Nov 24];17(2):165–9. Available from: http://content.wkhealth.com/linkback/openurl?sid=WKPTLP:landingpage&an=00075198-201104000-00014
10. Knaus WA, Draper EA, Wagner DP, Zimmerman JE. APACHE II: a severity of disease classification system. Crit Care Med [Internet]. 1985 Oct [cited 2018 Aug 30];13(10):818–29. Available from: http://www.ncbi.nlm.nih.gov/pubmed/3928249
11. Gall JR, Lemeshow S, Saulnier F. A New Simplified Acute Physiology Score (SAPS II) Based on a European/North American Multicenter Study. JAMA J Am Med Assoc. 1993;
12. Kamath PS, Wiesner RH, Malinchoc M, Kremers W, Therneau TM, Kosberg CL, et

al. A model to predict survival in patients with end-stage liver disease. Hepatology. 2001;

13. Kim WR, Biggins SW, Kremers WK, Wiesner RH, Kamath PS, Benson JT, et al. Hyponatremia and mortality among patients on the liver-transplant waiting list. N Engl J Med. 2008;

14. Knaus WA, Draper EA, Wagner DP, Zimmerman JE. APACHE II: A severity of disease classification system. Crit Care Med. 1985;13(10):818–29.

15. Malinchoc M, Kamath PS, Gordon FD, Peine CJ, Rank J, ter Borg PCJ. A model to predict poor survival in patients undergoing transjugular intrahepatic portosystemic shunts. Hepatology. 2000 Apr;31(4):864–71.

16. Moreau R, Jalan R, Gines P, Pavesi M, Angeli P, Cordoba J, et al. Acute-on-chronic liver failure is a distinct syndrome that develops in patients with acute decompensation of cirrhosis. Gastroenterology. 2013 Jun;144(7):1426–37, 1437.e1-9.

17. Cheatham ML, Malbrain MLNG, Kirkpatrick A, Sugrue M, Parr M, De Waele J, et al. Results from the international conference of experts on intra-abdominal hypertension and abdominal compartment syndrome. II. Recommendations. In: Intensive Care Medicine. 2007. p. 951–62.

18. Malbrain MLNG, Cheatham ML, Kirkpatrick A, Sugrue M, Parr M, De Waele J, et al. Results from the International Conference of Experts on Intra-abdominal Hypertension and Abdominal Compartment Syndrome. I. Definitions. In: Intensive Care Medicine [Internet]. Intensive Care Med; 2006 [cited 2020 Aug 24]. p. 1722–32. Available from: https://pubmed.ncbi.nlm.nih.gov/16967294/

19. De Laet IE, Malbrain MLNG, De Waele JJ. A Clinician’s Guide to Management of

Intra-Abdominal Hypertension and Abdominal Compartment Syndrome in Critically Ill Patients [Internet]. Vol. 24, Critical Care. BioMed Central Ltd.; 2020 [cited 2020 Sep 30]. p. 97. Available from: https://ccforum.biomedcentral.com/articles/10.1186/s13054-020-2782-1

20. Nadim MK, Durand F, Kellum JA, Levitsky J, O'Leary JG, Karvellas CJ, et al. Management of the critically ill patient with cirrhosis: A multidisciplinary perspective. In: Journal of Hepatology [Internet]. Elsevier B.V.; 2016 [cited 2020 Sep 30]. p. 717–35. Available from: https://pubmed.ncbi.nlm.nih.gov/26519602/

21. Al-Dorzi HM, Tamim HM, Rishu AH, Aljumah A, Arabi YM. Intra-abdominal pressure and abdominal perfusion pressure in cirrhotic patients with septic shock. Ann Intensive Care. 2012 Jul;2012:S4.

22. Arabi YM, Aljumah A, Dabbagh O, Tamim HM, Rishu AH, Al-Abdulkareem A, et al. Low-dose hydrocortisone in patients with cirrhosis and septic shock: A randomized controlled trial. CMAJ. 2010 Dec 14;182(18):1971–7.

23. De Laet I, Citerio G, Malbrain MLNG. The influence of intra-abdominal hypertension on the central nervous system: Current insights and clinical recommendations, is it all in the head? In: Acta Clinica Belgica. 2007. p. 89–97.

24. Deeren DH, Dits H, Malbrain MLNG. Correlation between intra-abdominal and intracranial pressure in nontraumatic brain injury. Intensive Care Med. 2005 Nov;31(11):1577–81.

25. Youssef AM, Jahromi AH, Vijay CG, Granger DN, Alexander JS. Intra-abdominal hypertension causes reversible blood-brain barrier disruption. In: Journal of Trauma and Acute Care Surgery. J Trauma Acute Care Surg; 2012. p. 183–8.

26. Dabrowski W. Changes in intra-abdominal pressure and central venous and brain venous blood pressure in patients during extracorporeal circulation. Med Sci Monit [Internet]. 2007 Dec [cited 2020 May 29];13(12):CR548-54. Available from: http://www.ncbi.nlm.nih.gov/pubmed/18049434

27. Citerio G, Vascotto E, Villa F, Celotti S, Pesenti A. Induced abdominal compartment syndrome increases intracranial pressure in neurotrauma patients: A prospective study. Crit Care Med. 2001;29(7):1466–71.

28. Marinis A, Argyra E, Lykoudis P, Brestas P, Theodoraki K, Polymeneas G, et al. Ischemia as a possible effect of increased intra-abdominal pressure on central nervous system cytokines, lactate and perfusion pressures. Crit Care. 2010 Mar 15;14(2).

29. Wilson MH. Monro-Kellie 2.0: The dynamic vascular and venous pathophysiological components of intracranial pressure [Internet]. Vol. 36, Journal of Cerebral Blood Flow and Metabolism. Nature Publishing Group; 2016 [cited 2020 Apr 23]. p. 1338–50. Available from: http://www.ncbi.nlm.nih.gov/pubmed/27174995

30. Depauw PRAM, Groen RJM, Van Loon J, Peul WC, Malbrain MLNG, De Waele JJ. The significance of intra-abdominal pressure in neurosurgery and neurological diseases: a narrative review and a conceptual proposal. Vol. 161, Acta Neurochirurgica. Springer-Verlag Wien; 2019. p. 855.

31. Weil D, Levesque E, McPhail M, Cavallazzi R, Theocharidou E, Cholongitas E, et al. Prognosis of cirrhotic patients admitted to intensive care unit: a meta-analysis. Ann Intensive Care. 2017 Dec;7(1):33.

32. Pereira R, Bagulho L, Cardoso FS. Acute-on-chronic liver failure syndrome -

clinical results from an intensive care unit in a liver transplant center. Rev Bras Ter intensiva [Internet]. 2020 [cited 2020 Jun 25];32(1):49–57. Available from: https://www.ncbi.nlm.nih.gov/pmc/articles/PMC7206960/

33. Verdelho M, Perdigoto R, Machado J, Mateus É, Marcelino P, Pereira R, et al. Acute-on-Chronic Liver Failure: A Portuguese Single-Center Reference Review. GE Port J Gastroenterol [Internet]. 2018 Jan [cited 2019 Mar 20];25(1):18–23. Available from: https://www.karger.com/Article/FullText/478988

34. Cardoso FS, Abraldes JG, Sy E, Ronco JJ, Bagulho L, Mcphail MJ, et al. Lactate and number of organ failures predict intensive care unit mortality in patients with acute-on-chronic liver failure. Liver Int [Internet]. 2019 Mar 19 [cited 2019 Aug 14];39(7):liv.14083. Available from: http://www.ncbi.nlm.nih.gov/pubmed/30825255

35. Jalan R, Saliba F, Pavesi M, Amoros A, Moreau R, Ginès P, et al. Development and validation of a prognostic score to predict mortality in patients with acute-on-chronic liver failure. J Hepatol. 2014 Nov;61(5):1038–47.

36. Levesque E, Hoti E, Jiabin J, Dellamonica J, Ichai P, Saliba F, et al. Respiratory impact of paracentesis in cirrhotic patients with acute lung injury. J Crit Care. 2011 Jun;26(3):257–61.

37. Phillip V, Saugel B, Ernesti C, Hapfelmeier A, Schultheiß C, Thies P, et al. Effects of paracentesis on hemodynamic parameters and respiratory function in critically ill patients. BMC Gastroenterol. 2014 Jan 27;14(1):18.

38. Mayr U, Karsten E, Lahmer T, Rasch S, Thies P, Henschel B, et al. Impact of large volume paracentesis on respiratory parameters including transpulmonary pressure and on transpulmonary thermodilution derived hemodynamics: A

prospective study. PLoS One. 2018 Mar 1;13(3).

39. Mayr U, Fahrenkrog-Petersen L, Batres-Baires G, Herner A, Rasch S, Schmid RM, et al. Large-volume paracentesis effects plasma disappearance rate of indo-cyanine green in critically ill patients with decompensated liver cirrhosis and intraabdominal hypertension. Ann Intensive Care. 2018 Dec 1;8(1).

40. Umgelter A, Reindl W, Wagner KS, Franzen M, Stock K, Schmid RM, et al. Effects of plasma expansion with albumin and paracentesis on haemodynamics and kidney function in critically ill cirrhotic patients with tense ascites and hepatorenal syndrome: A prospective uncontrolled trial. Crit Care. 2008 Jan 15;12(1).

41. Pereira, Rui (Hospital Curry Cabral C. Continuous Passive Paracentesis for Intra-abdominal Hypertension - Full Text View - ClinicalTrials.gov [Internet]. [cited 2020 May 29]. Available from: https://clinicaltrials.gov/ct2/show/NCT04322201

Declarations

Ethics approval and consent to participate. The Institutional Ethics Committees waived the need for individual informed consent for this observational study. All study procedures followed the principles of the Declaration of Helsinki.

Consent for publication. Not applicable.

Availability of data and materials. The datasets used and/or analyzed during the current study are available from the corresponding author on reasonable request.

Competing interests. The authors declare to have no conflicts of interest to disclose.

Funding. The authors declare this study received no funding.

Authors' contributions. Rui Pereira designed the study, acquired data, performed analysis and drafted the manuscript. Maria Buglevski and Stijn Blot acquired data. Rui Perdigoto, Paulo Marcelino, Faouzi Saliba, Stijn Blot and Joel Starkopf have contributed with interpretation of data and manuscript revision.

All the authors approved the submitted version and have agreed both to be personally accountable for the author's own contributions and to ensure that questions related to the accuracy or integrity of any part of the work, even ones in which the author was not personally involved, are appropriately investigated, resolved, and the resolution documented in the literature.

Acknowledgements. The authors of this study would like to acknowledge Dr. Nuno Germano, the Intensive Care Medicine nursing and medical staff at Hospital de Curry Cabral and Tartu University Hospital, the ESICM Trials Group Project collaborators in the “Abdominal

Sepsis Study: Epidemiology of Etiology and Outcome” (Supplemental file 1) and Centro de Investigação at Centro Hospitalar Universitário Lisboa Central for their contribution.

Figure 1: Patient flowchart. Abbreviations: AbSeS – Abdominal Sepsis Study: Epidemiology of Etiology and Outcome; IAPm – Intra-Abdominal Pressure measure

Figure 2: Maximum intra-abdominal pressure and mortality rates in critically ill liver cirrhotic patients. Abbreviations: IAH – Intra-abdominal hypertension; ICU – intensive care unit.

Figure 3: Mean intra-abdominal pressure and mortality rates in critically ill liver cirrhotic patients. Abbreviations: IAH – Intra-abdominal hypertension; ICU – intensive care unit.

Supplemental figure 1: Intra-abdominal hypertension grades distribution assessed by maximum and mean values in critically ill cirrhotic patients. Abbreviations: IAH – Intraabdominal Hypertension; IAP – Intra-abdominal Pressure.

Supplemental figure 2. Number of organ failures at ICU admission and mortality rates in liver cirrhotic patients. Abbreviations: OF – organ failure; ICU – intensive care unit.

Click here to access/download;Figure;Table 1.docx

Table 1: Baseline characteristics of liver cirrhosis patients in Intensive Care and 28-day mortality

	Total patients (n=95)	Non-survivors (n=44)	Survivors (n=50)	p value
Age (years)	56.7 ± 1.3	57.2 ± 11.0	56.3 ± 10.0	0.5
Male gender (n, %)	75 (79)	41 (55)	34 (45)	0.9
Etiology of liver cirrhosis (n, %) [a]				
Alcohol	43 (45)	26 (60)	17 (40)	
Alcohol plus HCV	6 (10)	3 (33)	6 (67)	0.5
HCV	6 (6)	3 (50)	3 (50)	
Precipitating event (n, %) [b]				
Infection	25 (26)	14 (56)	11 (44)	0.8
Bleeding	20 (21)	12 (60)	8 (40)	0.6
CCS (n=64)	5.4 ± 2.1	5.6 ± 2.1	5.1 ± 2.1	0.4
MELD (n=87)	26.2 ± 9.9	30.4 ± 30.4	21.6 ± 7.5	<0.001
MELD-Na (n=87)	27.7 ± 9	31.4 ± 9.0	23.7 ± 7.1	<0.001
APACHE II (n=88)	25.3 ± 10.1	28.2 ± 11.3	22.1 ± 7.7	0.004
SAPS II (n=89)	48.5 ± 15.3	54.7 ± 15.1	41.5 ± 12.5	<0.001
CLIF-SOFA (n=87)	12.8 ± 3.6	13.7 ± 3.7	11.8 ± 3.3	0.01
SOFA (n=83)	11.3 ± 3.4	11.7 ± 3.4	10.8 ± 3.4	0.2
Organ failure (n=87)	2.2 ± 1.2	2.5 ± 1.3	1.9 ± 1.0	0.01
Ascites (n=64) (n, %)	59 (92)	32 (73)	27 (54)	0.4
West-Haven score [IQR]	1.0 [0-3]	1.0 [0-3]	0.0 [0-2]	0.2
GCS [IQR](n=88)	14 [8-15]	14.0 [8-15]	15.0 [8-15]	0.2
Ammonia (mmol/L) [IQR](n=34)	153 [104-237]	155 [120-221]	144 [74-258]	0.3
Hematocrit (%) (n=59)	24.4 ± 5.9	23.8 ± 5.8	25.1 ± 6.1	0.4
Leucocytes (10 x 10^9/mL) (n=94)	13.7 ± 8.6	15.9 ± 9.5	11.3 ± 7.0	0.01
Platelets (10 x 10^9/mL) (n=85)	118 ± 91	129 ± 80.1	106 ± 101	0.2
INR (n=87)	2.4 ± 1.3	2.9 ± 1.6	1.9 ± 0.6	<0.001
Bilirubin (mg/dl) (n=88)	8.5 ± 9.6	11.3 ± 10.6	5.4 ± 7.3	0.003
Creatinine (mg/dl) (n=88)	2.1 ± 1.5	2.3 ± 1.5	1.9 ± 1.5	0.2
Sodium (mEq/L) (n=95)	136 ± 7.9	136 ± 7.8	137 ± 8	0.5
C-reactive protein (mg/L) (n=58)	60.7 ± 60.1	55.8 ± 63.4	63.3 ± 58.9	0.6
Lactate (mmol/l) (n=58)	4.1 ± 4.3	6.6 ± 6.1	3.5 ± 3.4	0.004
PaO2/FiO2 (mmHg) (n=83)	285 ± 111	311 ± 130.3	262 ± 99	0.06
Vital organ support (n, %) [c]	81 (85)	42 (52)	39 (48)	0.8
Vasopressors	68 (71)	37 (54)	31 (46)	0.7
IMV	63 (66)	35 (56)	28 (44)	0.5
RRT	19 (20)	12 (63)	7 (37)	0.4
Maximum IAP (mmHg)	16 ± 5.7	17.3 ± 6.3	14.6 ± 4.7	0.02
Mean IAP (mmHg)	12.1 ± 4.1	13.0 ± 4.8	11.2 ± 2.8	0.03
ICU LOS	10.8 ± 11.3	8.7 ± 8.7	13.1 ± 13.4	0.07

Number (n) equals 95 and is presented with mean and standard deviation (±) unless otherwise stated.

[a] p value is provided for the comparison of "alcohol alone plus combined" versus all other liver cirrhosis etiologies.

[b] p value is provided for the comparison of bleeding or infection versus all other precipitating events.

[c] Vital organ support refers to single or combined vasopressor, IMV or RRT during the entire ICU stay.

Abbreviations: ACS - abdominal compartment syndrome; APACHE II - Acute Physiology and Chronic Health Evaluation II; CCS - Charlson Comorbidity Score; CLIF - Chronic Liver Failure; GCS - Glasgow Coma score; HCV - hepatitis C virus; IAP - intra-abdominal pressure; IAH - intra-abdominal hypertension; IAP - intra-abdominal pressure; ICU - intensive care unit; INR - international normalization ratio; IQR - interquartile range; LOS - length-of-stay; MELD - Model for End Stage Liver Disease; MELD-Na - Model for End Stage Liver Disease Sodium; SAPS II - Simplified Acute Physiology Score II; SD - standard deviation; SOFA - Sequential Organ Failure Assessment.

Table 2.

Click here to access/download;Figure;Table 2.docx

Table 2: Comparison of intra-abdominal pressure groups of critically ill liver cirrhotic patients.

	No IAH (n=17)			IAH overall (n=78)			p value	IAH grade I-II (n=56)			ACS (n=22)			*p* value
Age (years)	56.5	±	10.1	56.8	±	10.6	0.90	56.7	±	10.8	57.2	±	10.5	0.86
Male gender (n, %)	15.0		(88)	60.0		(77)	0.51	42.0		(75)	18.0		(82)	0.77
Etiology of cirrhosis (n, %) [a]														
Alcohol	10.0		(59)	33.0		(42)		24.0		(43)	9.0		(41)	
Alcohol plus HCV	3.0		(18)	6.0		(7.7)	0.06	4.0		(7.1)	2.0		(9.1)	1.00
HCV	0.0		(0)	6.0		(7.7)		3.0		(5.4)	3.0		(13.6)	
Precipitating event (n, %) [b]														
Infection	4.0		(24)	21.0		(27)	1.00	12.0		(21)	9.0		(41)	0.10
Bleeding	3.0		(18)	17.0		(22)	1.00	10.0		(18)	7.0		(32)	0.23
CCS (n=64)	4.9	±	2.0	5.5	±	2.1	0.40	5.3	±	2.3	5.8	±	2.0	0.45
MELD (n=87)	26.7	±	9.0	26.0	±	10.2	0.81	24.4	±	9.7	30.2	±	10.4	0.03
MELD-Na (n=87)	28.9	±	8.1	27.4	±	9.2	0.55	26.0	±	8.8	31.0	±	9.5	0.04
APACHE II (n=88)	27.5	±	15.2	24.8	±	8.7	0.33	24.7	±	9.1	24.9	±	7.7	0.95
SAPS II (n=89)	48.2	±	12.3	48.5	±	16.0	0.94	48.1	±	17.2	49.5	±	12.7	0.75
CLIF-SOFA (n=87)	12.8	±	3.1	12.8	±	3.8	0.98	12.9	±	3.9	12.6	±	3.5	0.74
SOFA (n=83)	10.4	±	2.8	11.4	±	3.5	0.28	11.7	±	3.5	10.8	±	3.4	0.34
Organ failure (n=87)	2.2	±	1.4	2.2	±	1.2	0.94	2.3	±	1.1	2.1	±	1.3	0.62
Ascites (n=64) (n, %)	11.0		(92)	48.0		(92)	1.00	30.0		(94)	18		(90)	0.63
West-Haven score [IQR]	0.0		[0-1]	1.0		[0-3]	0.04	1.0		[0-3]	1.0		[0-3]	0.34
GCS [IQR] (n=88)	15.0		[14-15]	14.0		[7-15]	0.03	14.0		[7-15]	15.0		[9-15]	0.14
Ammonia (mmol/L) [IQR](n=34)	150		[94-235]	155		[106-243]	0.97	157		[92-243]	147		[117-213]	0.78
Hematocrit (%) (n=59)	24.0	±	6.7	24.4	±	5.8	0.84	23.5	±	4.7	26.1	±	7.1	0.13
Leucocytes (10 x 10^9/mL) (n=94)	14.4	±	9.7	13.5	±	8.4	0.69	12.4	±	8.1	16.1	±	8.9	0.08
Platelets (10 x 10^9/mL) (n=85)	131	±	136	116	±	81	0.56	111	±	78	127	±	89	0.48
INR (n=87)	2.5	±	1.2	2.4	±	1.4	0.73	2.2	±	1.1	2.7	±	1.9	0.17
Bilirubin (mg/dl) (n=88)	7.4	±	7.4	8.7	±	10.1	0.63	7.7	±	9.4	11.4	±	11.4	0.16
Creatinine (mg/dl) (n=88)	2.0	±	1.4	2.1	±	1.6	0.78	2.0	±	1.6	2.5	±	1.6	0.28
Sodium (mEq/L) (n=95)	132	±	9	137	±	7	0.02	137	±	8	136	±	6	0.53
C-reactive protein (mg/L) (n=58)	73	±	65	56	±	60	0.36	51	±	49	70	±	83	0.23
Lactate (mmol/l) (n=58)	5.1	±	5.2	5.1	±	5.2	0.96	5.1	±	5.2	5.1	±	5.5	0.97
PaO2/FiO2 (mmHg) (n=83)	242	±	112	298	±	119	0.10	291	±	126	315	±	100	0.45
Vital organ support (n, %) [c]	15.0		(88)	66.0		(85)	1.00	51.0		(91)	15.0		(68)	0.31
Vasopressors	11.0		(65)	57.0		(73)	0.56	43.0		(77)	14.0		(64)	0.27
IMV	12.0		(71)	51.0		(65)	0.78	40.0		(71)	11.0		(50)	0.11
RRT	3.0		(18)	16.0		(21)	1.00	14.0		(25)	2.0		(9.1)	0.21
28-day mortality	7		(41)	43		(55)	0.42	26		(46)	17		(77)	0.02
ICU mortality	6		(35)	38		(49)	0.42	24		(43)	14		(64)	0.13
ICU LOS (days)	9.4	±	10.8	11.1	±	11.5	0.60	11.4	±	13.0	10.3	±	6.1	0.71

Number (n) equals 95 and is presented with mean and standard deviation (±) unless otherwise stated.

[a] p value is provided for the comparison of "alcohol alone plus combined" versus all other liver cirrhosis etiologies.

[b] p value is provided for the comparison of bleeding or infection versus all other precipitating events.

[c] Vital organ support refers to single or combined vasopressor, IMV or RRT during the entire ICU stay.

Abbreviations: ACS - abdominal compartment syndrome; APACHE II - Acute Physiology and Chronic Health Evaluation II; CCS - Charlson Comorbidity Score; CLIF - Chronic Liver Failure; GCS - Glasgow Coma score; HCV - hepatitis C virus; IAP - intra-abdominal pressure; IAH - intra-abdominal hypertension; IAP - intra-abdominal pressure; ICU - intensive care unit; INR - international normalization ratio; IQR - interquartile range; LOS - length-of-stay; MELD - Model for End Stage Liver Disease; MELD-Na - Model for End Stage Liver Disease Sodium; SAPS II - Simplified Acute Physiology Score II; SD - standard deviation; SOFA - Sequential Organ Failure Assessment.

Figure 1.

Click here to access/download;Figure;Figure 1 - Patient flowchart.docx

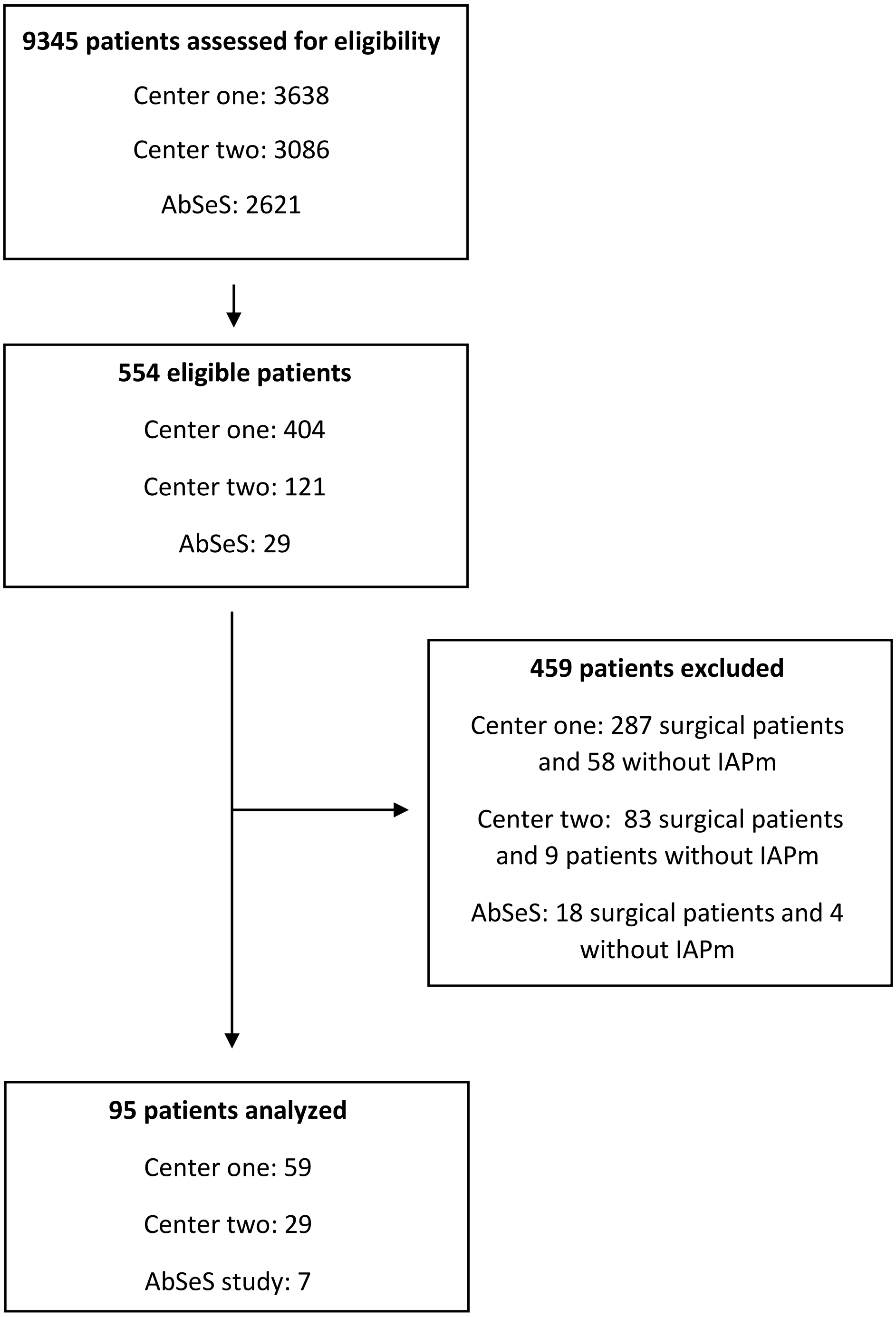

Figure 1: Patient flowchart.

Abbreviations: AbSeS – Abdominal Sepsis Study: Epidemiology of Etiology and Outcome; IAPm – Intra-Abdominal Pressure measure

Figure 2.

Click here to access/download;Figure;Figure 2 - Maximum IAP and mortality.docx

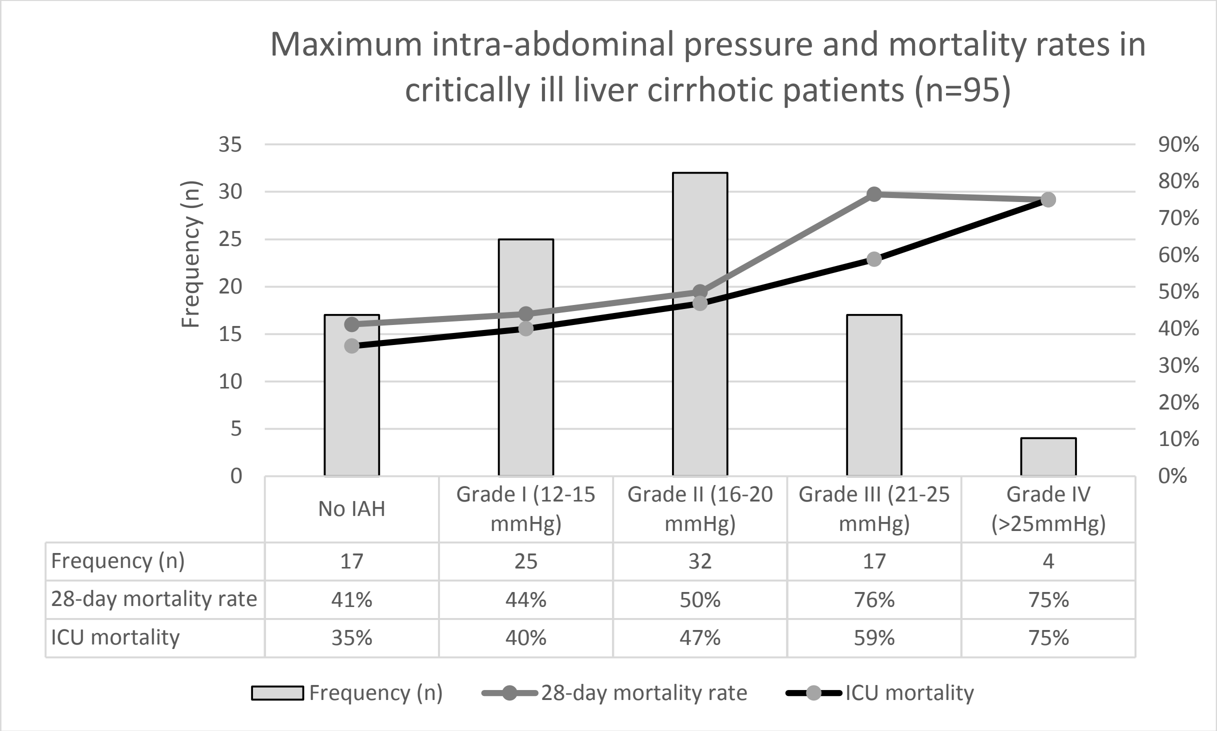

	No IAH	Grade I (12-15 mmHg)	Grade II (16-20 mmHg)	Grade III (21-25 mmHg)	Grade IV (>25mmHg)
Frequency (n)	17	25	32	17	4
28-day mortality rate	41%	44%	50%	76%	75%
ICU mortality	35%	40%	47%	59%	75%

Figure 2: Maximum intra-abdominal pressure and mortality rates in critically ill liver cirrhotic patients.

Abbreviations: IAH – Intra-abdominal hypertension; ICU – intensive care unit.

Figure 3.

Click here to access/download;Figure;Figure 3 - Mean IAP and mortality.docx

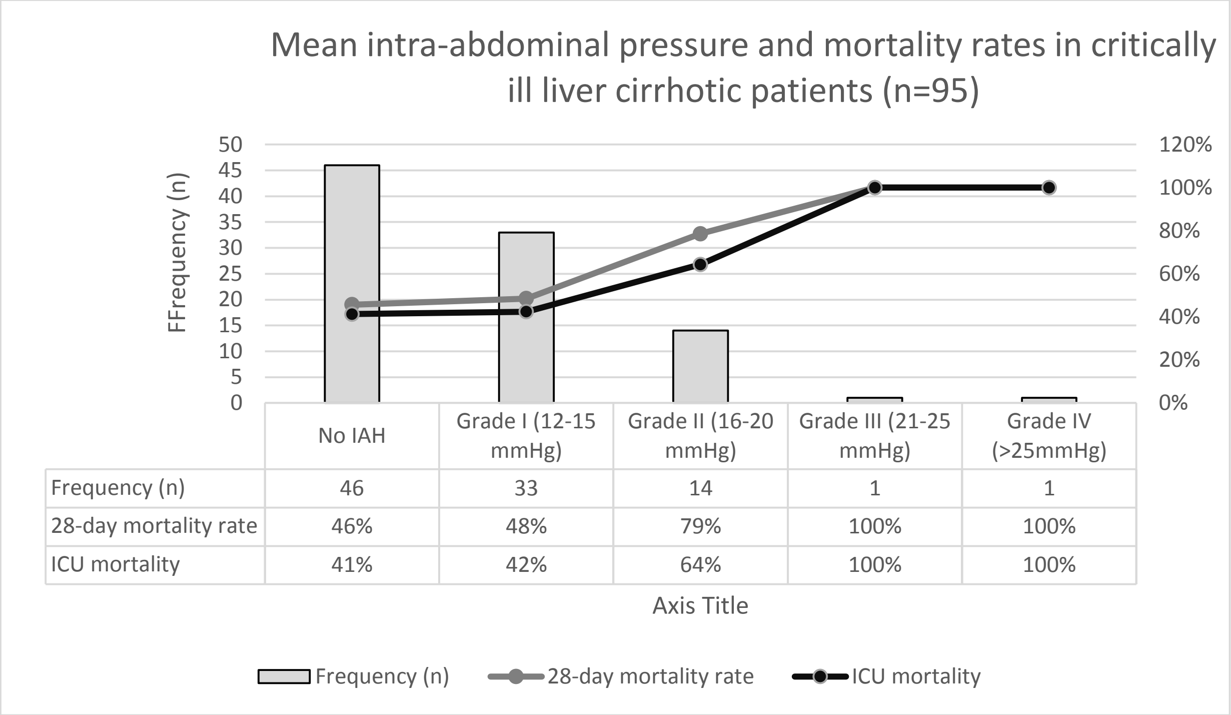

	No IAH	Grade I (12-15 mmHg)	Grade II (16-20 mmHg)	Grade III (21-25 mmHg)	Grade IV (>25mmHg)
Frequency (n)	46	33	14	1	1
28-day mortality rate	46%	48%	79%	100%	100%
ICU mortality	41%	42%	64%	100%	100%

Figure 3: Mean intra-abdominal pressure and mortality rates in critically ill liver cirrhotic patients.

Abbreviations: IAH – Intra-abdominal hypertension; ICU – intensive care unit.

Supplemental table 1

Click here to access/download
Supporting Information
Supplemental table 1.docx

Supplemental table 2

Click here to access/download
Supporting Information
Supplemental table 2.docx

Supplemental figure 1

Click here to access/download
Supporting Information
Supplemental figure 1 - IAP distributions.docx

Supplemental figure 2

Click here to access/download
Supporting Information
Supplemental figure 2 - Organ failure and mortality.docx

Click here to access/download
Supporting Information
Supplemental data file 1 - Collaborators AbSeS study.docx
