# Supplementary material for: Intra-abdominal hypertension and abdominal compartment syndrome in the critically ill liver cirrhotic patient–prevalence and clinical outcomes. A multicentric retrospective cohort study in intensive care
Source: PLoS One. 2021 May 13;16(5):e0251498. doi: 10.1371/journal.pone.0251498 (PMC8118291; doi:10.1371/journal.pone.0251498)
Supplement: Attachment — Submitted filename: Response to Reviewers.docx [file pone.0251498.s008.docx]

Response to Reviewers

Dear Editors and Reviewers

I would like to thank for the feedback and the constructive remarks, as well as the opportunity to improve the manuscript.

All posed questions by the reviewers have been addressed and highlighted in this document and a major revision has been made in “Revised Manuscript with track changes” and “Manuscript”, along with the remaining support material, as instructed.

I hope these changes have resulted in a significant improvement of the manuscript.

Looking forward to your reply.

Kind regards

Rui Pereira

Lisboa, 23/04/2021

Academic Editor:

Thank you for your kind feedback and attention.

1. Please ensure that your manuscript meets PLOS ONE's style requirements, including those for file naming. I apologize for this incorrection, and needed adjustments have been made to submission to meet the style requirements.

2. We note that you have indicated that data from this study are available upon request. PLOS only allows data to be available upon request if there are legal or ethical restrictions on sharing data publicly.

We have made the data available and submitted as a supporting file (“.sav”, SPSS® software).

3. Please include your tables as part of your main manuscript and remove the individual files. Please note that supplementary tables should remain as separate "supporting information" files.

These changes have been incorporated to the manuscript as file names have been adjusted.

4. Your ethics statement should only appear in the Methods section of your manuscript. If your ethics statement is written in any section besides the Methods, please move it to the Methods section and delete it from any other section.

These changes have been made, and Ethics statement is only included in Methods and Materials section.

Reviewer #1:

Introduction:

1.

*“The reported prevalence of IAH/ACS in mixed medical and surgical ICU patients reach 59% and 8%, respectively.” – It is unclear as to what these figures are referring.*

Thank you for your comment. The mentioned sentence in Introduction section has been split into two sentences for improved clarity.

*The prevalence of IAH and ACS are two different entities. It would be helpful here to define IAH and ACS. The prevalence of IAH in this population is much higher than that of ACS. As per the WSACS consensus statement, IAH is an intra-abdominal pressure >12mmHg, whilst ACS is >20mmHg with organ dysfunction. This should be clearly defined in the text.*

Definitions, measures and clinical management of IAP have been highlighted in a specific paragraph in Methods and Materials section.

2.

*Although there is discussion of the treatment of ACS by paracentesis, it would be useful to clarify what the clinical sequelae are of the condition, For example, renal impartment, ileus and intestinal failure may all result from IAH and should be included in order to place the article in the clinical context.*

A brief description of the complications of IAH with ACS, namely, acute organ dysfunctions, was added to the Introduction section.

3.

*What is the operative intervention rate and mortality rate of IAH/ACS?*

The mortality rate of mixed (surgical and medical) patients with IAH ranged between 25-59%, this data has been included in the Introduction section.

The operative intervention rate and mortality rate in IAH/ACS are very dependent on the specific underlying surgical pathology (i.e. ruptures abdominal aortic aneurism, trauma, etc…) and it is very difficult to accurately provide an overall rate. In our study all patients were of the medical type, specifically, liver cirrhosis patients with acute medical (non-surgical) decompensation, therefore there were no operative interventions in our cohort. Reference to the “non-surgical” type of patients in this study has been highlighted in Introduction and Methods and Materials sections.

Materials and Methods

1.

*It is useful to include multicentre work in such studies and this is to be recognised as a valuable approach. However there appears to be a discrepancy in the length of time over which the data was collected (October 2016-2019 in the first centre, 2010-2018 in the second). Why is this? Was this due to a change in practice at these centres or due to data availability?*

The discrepancy between data collection dates is due to data availability, since measure of IAP became a standard procedure in the second center in 2010 and only in 2016 for the first center. Regarding clinical practice, major clinical guidelines for IAP/ACS have been published in 2006 and revised in 2013 without changes to clinical pratice.(1,2) The 2013 WSACS consensus definitions and clinical guidelines only changed the definitions regarding “*removal of the filtration gradient definition, and the proposal of definitions for the poly-compartment syndrome, abdominal compliance, the open abdomen, and lateralization of the abdominal musculature”* (in *Supplementary material 5. Elaboration of the Changes and Additions to the Previous 2006 Consensus Definitions*)”.(2) Therefore guidelines and clinical practice throughout the study period have remained largely unchanged regarding the management of IAH and ACS. This has now been stated in the Methods and Material section as “*The study period length was determined by data availability while clinical guidelines remained largely unchanged regarding the management of IAH/ACS*.”

1. Malbrain ML, Cheatham ML, Kirkpatrick A, Sugrue M, Parr M, De Waele J, Balogh Z, Leppaniemi A, Olvera C, Ivatury R, D’Amours S, Wendon J, Hillman K, Johansson K, Kolkman K, Wilmer A (2006) Results from the international conference of experts on intra-abdominal hypertension and abdominal compartment syndrome. I. Definitions. Intensive Care Med 32:1722–1732
2. Kirkpatrick, A.W., Roberts, D.J., De Waele, J. et al. Intra-abdominal hypertension and the abdominal compartment syndrome: updated consensus definitions and clinical practice guidelines from the World Society of the Abdominal Compartment Syndrome. Intensive Care Med 39, 1190–1206 (2013). https://doi.org/10.1007/s00134-013-2906-z

2.

*“The Institutional Ethics Committees waived the need for individual informed consent for this observational study.” – I am assuming the ethics committees for the two institutions named, could you clarify this in the text?*

I appreciate your remark. The Institutional Ethics Committees from both participating centers waived the need for individual informed consent, namely, Centro Hospitalar Universitario Lisboa Central (center one) reference number 632/2018 and University of Tartu (center two) reference number 264/T-4). The statement has been clarified to specify both centers.

3.

*You excluded patients if they had an ICU admission for ‘surgical reasons’. Is this purely abdominal surgery? This would have a different confounding effect on the prevalence of IAH compared to, for example, orthopaedic surgery or trauma. This needs clarification.*

In our study all patients are liver cirrhotic with acute medical decompensation, as these represent a very specific set of patients, indeed only of the medical type. Therefore, all types of surgical patients were excluded from this cohort and accounted for in Figure 1 “*Patient flowchart*”. Clarification has been made in "*The exclusion criteria were absence of any IAP recordings and/or any type of surgical patients, since most surgical ICU patients present anatomical changes to the chest and/or abdominal wall, interfering with the reference normal range for IAP differs. Therefore, this cohort was restricted to liver cirrhotic patients with acute medical (non-surgical) decompensation”*.

4.

The overall structures of the methods section is a little difficult to follow. I would consider restructuring it into baseline variables, IAP assessment, clinical management, statistical analysis.

The Methods and Materials section has been, accordingly, restructured into settings and design, baseline variables, IAP assessment, clinical management and statistical analysis.

5.

The methods of statistical analysis appear appropriate.

Results

1.

I am not clear on the data in figure one. You have stated 554 eligible patients, but then in the next step on the flowchart excluded 459 patients. Would it not be better to say than 554 developed IAH? Or do you mean there were 554 cirrhotic patients? This really isn’t clear and needs to be amended.

Thank you for your rightful comment. Amendments for clarification have been made in the Methods (eligibility, inclusion/exclusion criteria), Results section and in Figure 1.

*“A total of 9,345 ICU admissions were screened, of which 554 were adult liver cirrhosis patients in their index ICU admission; and 459 were excluded either due to surgical patient type and/or absence of IAP measure. Ninety-five patients were included in this analysis.”*

2.

Have you considered an analysis of the timing of IAH measurement? For instance, it would be helpful to understand if mean IAP was related to the length of stay in the ICU.

Thank you for these questions.

Indeed, we have considered the analysis of the timing of IAH measurement. Unfortunately, overall data could not provide precise IAP measurement timing nor trends during ICU stay, this is now included in the Limitations section, and the ongoing study COPPTRIAHL will address this issue. Regarding mean IAP or maximum IAP and ICU length-of-stay we did not find significant correlation (Pearson coefficient) between these variables, and this has now been stated in Results. Additionally, regarding mean IAP, we have added to the Discussion “*that mean IAP presented a narrow IAH grade I and II distribution when compared to maximum IAP (Supplemental figure 1). This difference could have been partly due to the clinical management of IAP reflecting a sustained reduction of IAH throughout the ICU stay. Furthermore, it could indicate that maximum IAP was a better marker of severity of the underlying liver cirrhosis with portal hypertension and ascites, although our data does not permit us to draw such conclusions.*”

3.

It is interesting that alcoholic cirrhosis was an independent risk factor. Did you undertake an analysis also of the underlying aetiology of ICU admission for worsening of cirrhosis?

Thank you very much for these observations.

Our data showed that over half of the study population had alcohol related cirrhosis, and the remaining etiologies were either scarce in number or unspecified preventing us to elaborate on this aspect.

Regarding the precipitating events of decompensated cirrhosis leading to ICU admission we have better described these in the Tables and Results section and, indeed, after looking at the data once more, when we divided precipitating events into “Infection/Bleeding/Others” categories we found that the subgroup “Infection” did have statistical significance after multivariate analysis. This has been adjusted in the Results section and tables and commented in the Discussion section.

As an addition, the total number of Organ Failures at ICU admission described in Table 1 and 2 was now included in Results section, for better characterization of these patients at ICU admission.

I would also like to know how many patients had paracentesis, as this would likely be a significant factor in prevention of ACS.

We recognize this important question although in this study we could not get adequate data to properly answer this question, this has been stated in the Limitations section. Even so, for the purpose of this study of IAH, and regardless of any measure for reducing it, such as paracentesis, it can be argued that our focus was the actual prevalence and the association of IAH with clinical outcomes, rather than the effect of a specific attitude or therapy. The ongoing study COPPTRIAHL will address therapeutic paracentesis adequately.

Discussion

4.

I think the conclusions you have drawn from your results are reasonable. However, it should be noted that the lack of abdominal perfusion pressure is a major limitation. Presumably this could have bene obtained, and it may be worth looking at these patients again if the mean arterial pressure is available (although I understand that not all patients will have invasive blood pressure monitoring.

We agree this is an important issue, and we recognize it in Limitations section. The available data did not allow us to adequately analyze abdominal perfusion pressure at ICU admission nor follow its trends during ICU stay, therefore, we opted not to present it nor draw any conclusions. Once again, we acknowledge this issue in Limitations section and have the ongoing COPPTRIAHL clinical trial that will address this issue.

5.

Certainly, your results are in line with other studies looking at cirrhosis as a risk factor for abdominal compartment syndrome, which as you have quite rightly identified has a significantly higher prevalence in this population.

Reviewer #2:

- What is the standard IAP measurement in this study? Is it the same technique in both centers in this study?

This study complied with the clinical guidelines from the World Society of Abdominal Compartment Syndrome for IAP assessment and definitions, this has now been better described in a specific paragraph in Methods and Materials section. The normal range of IAP in critically ill patients should be 5-7 mmHg using an indirect trans-bladder measure with 25cc saline infusion and zero pressure reference point in the mid-axillary line.

- Can you define the accurate study month in the second center?

Thank you for the question. There was an incidental mistake regarding the beginning year of the screening period for the second center which is set between 01/01/200**9** and 31/12/2018. This has been corrected and detailed in Methods and Material section.

- What is the frequency of IAP measurement? and how to calculate the data (cmH2O to mmHg) in this study?

For this retrospective study, the baseline frequency was trice daily measurements of IAP, although, it must be openly said that in center one not all patients had 3 measures for all the days and in center two, as well as, the AbSeS study data was collected and resulted in s simple maximum and mean values for the entire ICU stay, as used in this study. This is described in Results. The standard units of IAP measurement are expressed in mmHg, so it can be directly interpreted as with blood pressure in clinical practice.

- IQR should be represent in (Q1, Q3) not the range.

Thank you for the remark. This has been acknowledged and changed throughout the manuscript and tables

- This study reported very high prevalence of IAH. It may from the measurement method or recording technique. Please define the accurate method.

Indeed, the very high prevalence of IAH was expected in this population of liver cirrhosis patients with ascites, particularly due to critical illness, intensive fluid therapy and increased leakage into third space ascites. This high prevalence of IAH was previously described in a study by Al-Dorzi, specifically on septic shock patients, as mentioned in the Discussion section. Additionally, a patient selection bias, which could have increased the reported prevalence, was minimized given the fact that all the centers were routinely collecting IAP data in liver disease patients, regardless of this study.

In our study the measurement method for IAP was standardized by international clinical guidelines and avoided some common pitfalls. This has now been highlighted in a specific paragraph (“Intra-abdominal pressure assessment…”) in Methods and Materials section.

- In tables, the classification of the data should be made to make it easier to read such as demographic data, cirrhosis parameters and scores, clinical signs, laboratory, measurement data, and result.

We have introduced line breaks on the tables to clearly separate the subsets of data.

- In patient flowchart, this study defines as a cohort study, the analyzed cases should be classified in to groups which the investigator would like to compare (IAH, ACS, no IAH?).

The patient flowchart (Figure 1) has now been updated to include the study groups (no IAH, IAH and ACS).

- In figures, categorized of IAH/ACS is based on the maximum IAP, not the mean IAP that why the frequency of the data of figure 2 and 3 is not same. Please revise these figures.

Our purpose in displaying both figures 2 and 3 was to provide the reader with distinct images between the distributions of IAH grades considering either maximum or mean IAP during ICU stay and their respective mortality rates. Perhaps our intention was not clear. We have clarified this in Results section: “The IAH grade distribution of maximum and mean IAP during ICU stay are, respectively, shown in Figure 2 and 3, and compared in Supplemental Figure 1*”*

- You can make this article easier to read by reducing paragraphs and unnecessary words.

Thank you for feedback on this topic. Since our native language is not English we have used a proofreading service (Cambridge Proofreading LLC, 11th Sep. 2020), for which we can provide a certificate. Nonetheless, given the structural changes made since, we continuously strived to improve the overall manuscript. The present major revision has been carried out to improve the ease of reading and understanding. I hope it has significantly improved.

Thank you very much for your time and attention.

Kind regards

Rui Pereira
